# In Situ Reconstructing NiFe Oxalate Toward Overall Water Splitting

**DOI:** 10.1002/advs.202408754

**Published:** 2024-10-03

**Authors:** Zhen Zhang, Xiaoyu Ren, Wenyuan Dai, Hang Zhang, Zhengyin Sun, Zhuang Ye, Ying Hou, Peizhi Liu, Bingshe Xu, Lihua Qian, Ting Liao, Haixia Zhang, Junjie Guo, Ziqi Sun

**Affiliations:** ^1^ Key Laboratory of Interface Science and Engineering in Advanced Materials Ministry of Education College of Materials Science and Engineering Taiyuan University of Technology Taiyuan 030024 P. R. China; ^2^ Materials Institute of Atomic and Molecular Science Shaanxi University of Science &Technology Xi'an 710021 P. R. China; ^3^ School of Physics Huazhong University of Science and Technology Wuhan 430074 P. R. China; ^4^ School of Mechanical Medical and Process Engineering Queensland University of Technology George Street Brisbane QLD 4000 Australia; ^5^ School of Chemistry and Physics Queensland University of Technology Brisbane QLD 4000 Australia; ^6^ Centre for Materials Science Queensland University of Technology 2 George Street Brisbane 4000 Australia

**Keywords:** amorphous catalysts, deep reconstruction, in situ Raman, overall water splitting, oxalates

## Abstract

Surface reconstruction plays an essential role in electrochemical catalysis. The structures, compositions, and functionalities of the real catalytic species and sites generated by reconstruction, however, are yet to be clearly understood, for the metastable or transit state of most reconstructed structures. Herein, a series of NiFe oxalates (Ni*_x_* Fe_1‐_ *_x_* C_2_O_4_, *x* = 1, 0.9, 0.7, 0.6, 0.5, and 0) are synthesized for overall water splitting electrocatalysis. Whilst Ni_x_Fe_1‐x_C_2_O_4_ shows great hydrogen evolution reaction (HER) activity, the in situ reconstructed Ni_x_Fe_1‐x_OOH exhibits outstanding oxygen evolution reaction (OER) activity. As identified by the in situ Raman spectroscopy and quasi‐in situ X‐ray absorption spectroscopy (XAS) techniques, reconstructions from Ni_x_Fe_1‐x_C_2_O_4_ into defective Ni_x_Fe_1‐x_OOH and finally amorphous Ni_x_Fe_1‐x_OOH active species (R‐Ni_x_Fe_1‐x_OOH) are confirmed upon cyclic voltammetry processes. Specifically, the fully reconstructed R‐Ni_0.6_Fe_0.4_OOH demonstrates the best OER activity (179 mV to reach 10 mA cm^−2^), originating from its abundant real active sites and optimal d‐band center. Benefiting from the reconstruction, an alkaline electrolyzer composed of a Ni_0.6_Fe_0.4_C_2_O_4_ cathode and an in situ reconstructed R‐Ni_0.6_Fe_0.4_OOH anode achieves a superb overall water splitting performance (1.52 V@10 mA cm^−2^). This work provides an in‐depth structure‐property relationship understanding on the reconstruction of catalysts and offers a new pathway to designing novel catalyst.

## Graphical abstract

This work discovers that the hydrogen evolution reaction (HER) active FeNi oxalates (Ni_x_Fe_1‐x_C_2_O_4_) undergo rapid in situ reconstruction into oxygen evolution reaction (OER) active FeNi oxyhydroxides (Ni_x_Fe_1‐x_OOH) upon electrochemical voltammetry cycles for reaching overall water splitting catalysis. This work provides an in‐depth structure‐property relationship understanding on the reconstruction of catalysts and offers a new pathway toward designing overall water splitting catalysts.

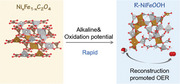

## 1 Introduction

The complex real water conditions and the associated alkalization with the consumption of H^+^ for hydrogen evolution, alkaline water electrolysis become a critical research focus for industrial scale green hydrogen production from water splitting.^[^ ^1^, ^2^, ^3^ ^]^ It is deemed as grant challengers that, however, the hydrogen evolution reaction (HER) becomes sluggish resulted from the additional water dissociation step to provide protons in alkaline environment, whilst the oxygen evolution reaction (OER) yet remains as a rate‐limiting step with high energy barriers for the O−H bond breaking and O−O bond formation.^[^ ^4^, ^5^, ^6^, ^7^, ^8^, ^9^ ^]^ Furthermore, to simplify the electrode structures and cell configurations for the practical application of electrochemical water splitting technology, bifunctional catalysts that exhibit simultaneous and satisfactory catalytic activity toward both HER and OER are also urgently demanded.^[^ ^10^, ^11^, ^12^, ^13^, ^14^, ^15^, ^16^, ^17^ ^]^ Therefore, exploring highly efficient bifunctional catalysts to overcome the unsatisfactory thermodynamics for alkaline HER and OER for overall water splitting is a matter of great urgency.

It is expected that the electrocatalysts could be maintained with robust stability and environmental durability. On the other hand, it has been widely accepted that the electrocatalysts would be undergone reconstruction under applied voltages with the formation of metastable or transit new structures.^[^ ^18^ ^]^ In some cases, the reconstructed structures perform the roles in regulating the adsorption and desorption behaviors of intermediates and enhancing the catalytic activities, which should be deemed as the authentic catalytic centers.^[^ ^19^, ^20^, ^21^, ^22^ ^]^ Based on this understanding, the reconstruction has been regarded as an effective strategy to design high‐performance electrocatalysts.^[^ ^23^, ^24^, ^25^, ^26^, ^27^, ^28^, ^29^, ^30^ ^]^ However, the active species formed via structure evolutions have been found to be extremely sensitive to the environment, which makes the accurately identification of the possible dynamic phase transition processes and the real active structures of the very trace of reconstructed catalysts very challenging.^[^ ^31^, ^32^ ^]^ It is very fortunate that the development of in situ and operando techniques enable the real‐time monitoring of dynamic structural transition under electrochemical cycles to be tangible.

In this work, a series of HER active Ni_x_Fe_1‐x_C_2_O_4_ (1, 0.9, 0.7, 0.6, 0.5, and 0) electrocatalysts is synthesized. It is interesting that Ni_x_Fe_1‐x_C_2_O_4_ exhibits outstanding OER catalytic performance after reconstruction. By employing in situ Raman spectroscopy and quasi‐in situ X‐ray absorption spectroscopy (XAS) techniques, a complete reconstruction from an initial Ni_x_Fe_1‐x_C_2_O_4_ structure to a final amorphous NiFeOOH structure (R‐Ni_x_Fe_1‐x_OOH) under oxidation potentials is monitored, which are the real OER catalytic species of the catalysts. The original Ni_0.6_Fe_0.4_C_2_O_4_ shows great HER activity with a low overpotential of 93 mV at 10 mA cm^−2^, and the completely reconstructed R‐Ni_0.6_Fe_0.4_OOH provides fascinating OER activity with a low overpotential of 179 mV to reach 10 mA cm^−2^. By constructing an alkaline water electrolyzer composed of original Ni_0.6_Fe_0.4_C_2_O_4_ as the cathode and reconstructed R‐Ni_0.6_Fe_0.4_OOH as the anode, overall water splitting can be achieved at a cell voltage of 1.52 V to reach 10 mA cm^−2^ and 1.77 V for a current density as high as 300 mA cm^−2^, demonstrating the bifunctionality and the practical application potential of the Ni_x_Fe_1‐x_C_2_O_4_ electrocatalyst. This work not only demonstrates a detailed study on in situ monitoring the dynamic structural reconstruction of electrocatalysts, but also provides a new approach to develop high‐efficient overall water splitting electrocatalysts.

## 2 Results and Discussion

### 2.1 Microstructure and Surface Chemistry of Ni_x_Fe_1‐x_C_2_O_4_ and Reconstructed NiFeOOH

**Figure 1:**
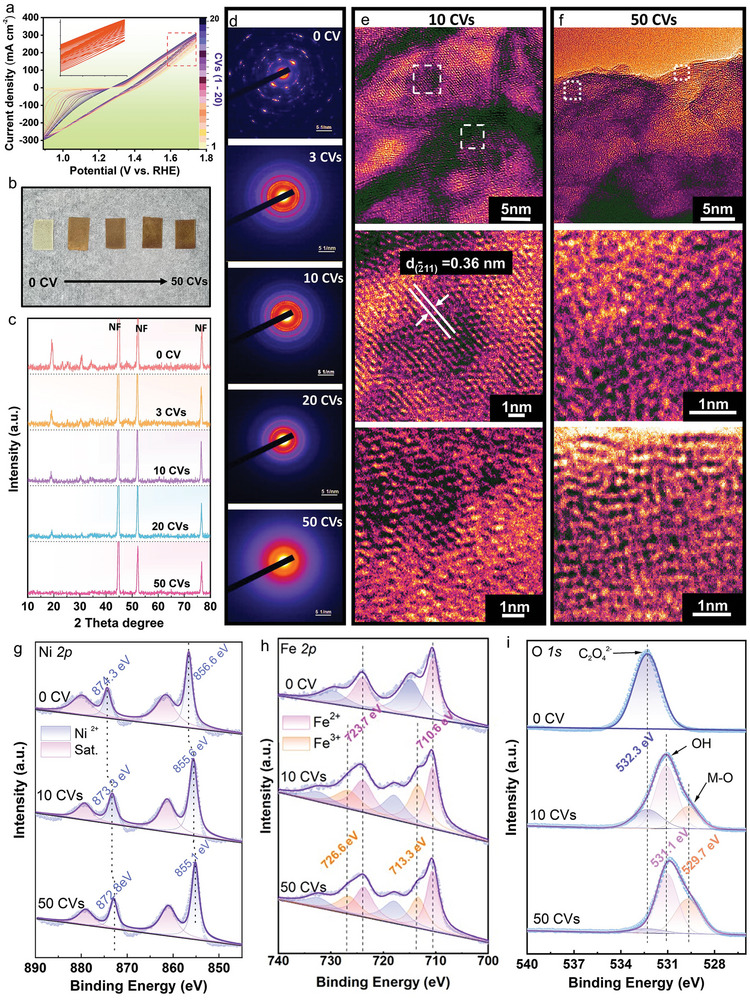
Microstructure and surface chemistry of surface reconstruction of Ni_0.6_Fe_0.4_C_2_O_4_ upon electrochemical cycles. a) Consecutive curves of Ni_0.6_Fe_0.4_C_2_O_4_ in 1 m KOH electrolyte, the inset in (a) is a larger version of the red dotted box. b) Optical images of Ni_0.6_Fe_0.4_C_2_O_4_ after different CV cycles. c) XRD patterns of Ni_0.6_Fe_0.4_C_2_O_4_ after different CV cycles. d) SAED of Ni_0.6_Fe_0.4_C_2_O_4_ after different CV cycles. e) HRTEM characterization of Ni_0.6_Fe_0.4_C_2_O_4_ after 10 CV cycles. h) HRTEM characterization of Ni_0.6_Fe_0.4_C_2_O_4_ after 50 CV cycles. XPS spectra of g) Ni 2p, h) Fe 2p and i) O 1s on Ni_0.6_Fe_0.4_C_2_O_4_ after different CV cycles. CVs scanned at a scan rate of 100 mV s^−1^.

NiFe‐oxalate (Ni_x_Fe_1‐x_C_2_O_4_) electrocatalysts on nickel foam (NF) was fabricated via a hydrothermal reaction (Figure S1, Supporting Information). To search the optimizing composition, a series of Ni_x_Fe_1‐x_C_2_O_4_ with an *x*‐value of 1, 0.9, 0.7, 0.6, 0.5, and 0 were synthesized (Figure S2, Supporting Information), which all are in rod‐like shapes (Figure S3, Supporting Information). Upon electrochemical cyclic voltammetry (CV) cycling, stepwise surface reconstructions from NiFe‐oxalates into final amorphous NiFeOOH were observed (**Figure** **1**a; Figure S4, Supporting Information). The electrochemical evaluations demonstrated that the electrocatalyst with an *x*‐value of 0.6 (Ni_0.6_Fe_0.4_ C_2_O_4_) presented the best performance. To simplify the description, we concentrate our focus on Ni_0.6_Fe_0.4_C_2_O_4_ except for those with specific notice. Figure 1a presents the CV curves carried out on Ni_0.6_Fe_0.4_C_2_O_4_ in 1 m KOH at a scan rate of 100 mV s^−1^. As the surface reconstruction can lead to significant increase of active sites, we used the electrochemical active surface area (ECSA) that correlated with the electrical double‐layer capacitor (*C*_dl_) to evaluate the extents of the reconstruction of Ni_0.6_Fe_0.4_C_2_O_4_. As shown in Figures S5 and S6 (Supporting Information), the as‐synthesized Ni_0.6_Fe_0.4_C_2_O_4_ had the lowest *C*_dl_ value (20.6 mF cm^−2^), which increased with the proceeding of the CV cycles and then saturated at 50 CVs. Therefore, the surface reconstruction of Ni_0.6_Fe_0.4_C_2_O_4_ is completed at 50 CV cycles. It is noted that the current density increased observably associated with gradual changes of color from yellowish‐green/chartreuse to brownish/umber for Ni_0.6_Fe_0.4_C_2_O_4_ with the CV cycles, confirming the occurrence of surface structure reconstructions (Figure 1b).

The typical X‐ray diffraction (XRD) patterns (Figure S7, Supporting Information) exhibits that the diffraction peaks of the original Ni_0.6_Fe_0.4_C_2_O_4_ located between those of NiC_2_O_4_ and FeC_2_O_4_. The energy dispersive X‐ray spectroscopy (EDX) mass percentages of Ni, Fe, C, and O elements in the as‐prepared Ni_0.6_Fe_0.4_C_2_O_4_ were identified to be 20.34%, 15.97%, 18.76%, and 44.92%, respectively, corresponding to a molar ration of Ni:Fe = 16:9 (ICP‐MS). It suggests that the NiFe‐oxalate is a homogenous solid solution of Ni/Fe oxalates without phase segregation, in combining with the corresponding EDX mapping (Figure S8d, Supporting Information). The well crystallized Ni_0.6_Fe_0.4_C_2_O_4_ grown on NF was in a rod morphology with diameters varying over 0.5 to 5 µm (Figure S8a–c, Supporting Information). The surface structure of the as‐prepared Ni_0.6_Fe_0.4_C_2_O_4_ was characterized by ex‐situ spectroscopy techniques. Typical vibrations ascribed to Ni─O, Fe─O, and C═O bonds were identified in the Raman spectrum (Figure S9, Supporting Information).^[^ ^33^, ^34^, ^35^, ^36^, ^37^ ^]^ In the X‐ray photoelectron spectroscopy (XPS) bands shown in Figure S10 (Supporting Information), the core levels corresponding to the states of Fe^2+^ and Ni^2+^ were observed in the Fe 2p and Ni 2p spectra, providing us a concrete support on our conjecture that obtained NiFe‐oxalate was a homogenous solid solution of Ni/Fe oxalates.

With the CV cycles performed in 1 m KOH at a scan rate of 100 mV s^−1^ without iR correction, no significant change of the main peaks (background peak of NF) on the XRD patterns (Figure 1c) was observed with the increase of the cycling number from 0 to 50 but the disappearance of the relative weak diffraction peaks, such as the one located at 20°, which might be resulted from the decrease of the crystallinity of the structure. In combing with the employment of high‐resolution transmission electron microscopy (HRTEM) observation (Figure 1e,f) and the corresponding selected area electron diffraction (SAED) characterizations (Figure 1d), it is very clear that the surface structures were gradually changed from well‐crystallized Ni_0.6_Fe_0.4_C_2_O_4_ structure (0 CVs) into a highly ordering structure (10 CVs) and finally an amorphous phase (50 CVs). The variation of the surface structure is associated with the change of valence states of the cations on the surface. Figure 1g–i presents the Quasi‐in situ XPS results. With the increase of CV cycles from 0 to 50, the core levels of Ni^2+^ shifted to lower binding energies (Figure 1g), suggesting the change of the Ni coordination. At high potential, NiFe oxalates were reconstructed into a R‐NiFeOOH or NiOOH phase, which, however, can be easily reduced back to a Ni(OH)_2_ phase in air, even if no reduction potential was applied.^[^ ^19^ ^]^ Therefore, no obvious Ni^3+^ signal could be observed on the XPS spectra collected externally on the samples (Figure 1g). The core levels of Fe 2p shifted from a Fe^2+^ (710.6 eV) state to more oxidized state close to Fe^3+^ (711.3 eV), properly ascribing to the transformation of FeC_2_O_4_ to FeOOH (Figure 1h).^[^ ^38^ ^]^ However, we should note that this structure achieved a complete reconstruction after 50 CVs (Figure 1c). Even after 50 CVs, a significant amount of Fe^2+^ still existed. Taking the HRTEM results into consideration, we can propose that the reconstruction should associate with the formation of FeOOH and oxygen vacancies: 2Fe^3+^→2Fe^2+^ + V_O_.^[^ ^39^ ^]^ Both the formation of oxyhydroxide and oxygen vacancies or the modulated oxygen‐linked microenvironment are highly desired in optimizing the adsorption energy of intermediates on the catalyst surface for reducing the reaction energy barrier and thus promoting OER activities.^[^ ^40^, ^41^, ^42^ ^]^ Meanwhile, the O 1s spectra (Figure 1i) showed the disappearance of C═O and the formation of M−O and lattice OH group (531‐532 eV) on the catalysts after 10 and 50 CVs, which confirm the alteration of O‐linked microenvironment.^[^ ^43^, ^44^, ^45^ ^]^ Furthermore, the chemical states located at 289.0 and 284.8 eV contributed by the C═O and C─C bonds in the C 1s spectra for pristine Ni_0.6_Fe_0.4_C_2_O_4_ (Figure S11, Supporting Information), disappeared after 10 CVs and 50 CVs, suggesting the possible transformation of Ni_0.6_Fe_0.4_C_2_O_4_ into NiFeOOH during the reconstruction.

### 2.2 In Situ/Quasi‐In Situ Characterization of Surface Reconstruction

**Figure 2:**
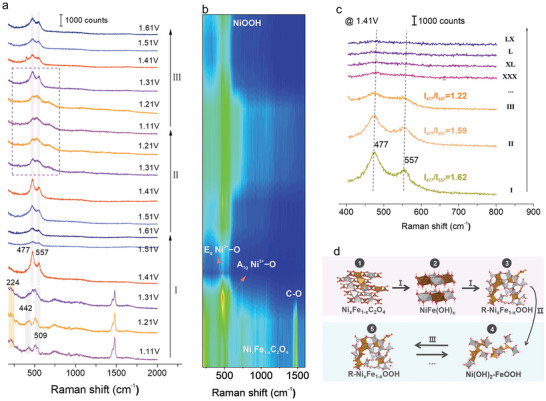
In situ Raman spectroscopy monitoring on surface reconstruction. a) In situ Raman spectra collected on Ni_0.6_Fe_0.4_C_2_O_4_ electrocatalysts in different processes. (Process I: potential ascending from 1.11 to 1.61 V; Process II: potential descending from 1.61 to 1.11 V; Process III: second potential ascending cycle; in 1 m KOH electrolyte at a scan rate of 1 mV s^−1^. b) The corresponding in situ Raman contour map. c) In situ Raman spectra collected at a potential of 1.41 V versus RHE in Process I, Process II, and Process III, and the 30th (XXX) cycle, the 40th (XL) cycle, the 50^th^ (L) cycle, and the 60^th^ (LX) cycle. (d) Phase transformation occurring in the reconstruction processes.

As what we concern, the real active sites on electrocatalysts are highly possible to be transit or metastable phases that are dynamically varied with the environment. To track these active sites, in situ or operando techniques are essential approaches for obtaining a correct mechanism understanding. The potential‐dependent phase changes of Ni_0.6_Fe_0.4_C_2_O_4_ were tracked by using an in situ Raman spectroscopy technique to monitor the real‐time surface chemistry during electrocatalysis. Figure S12 (Supporting Information) presents the configuration for the in situ electrochemical Raman examinations. **Figure** **2**a displays the in situ Raman spectra collected on the samples over the cycles of Process I: from 1.11  to 1.61 V, Process II: from 1.61  backward 1.11 V, and Process III: second cycle increase from 1.11  to 1.61 V. For a better presentation on the vibration variation in a range of 200–1000 cm^−1^ with the applied voltages, the changes of the vibration bands are also reflected in a corresponding contour map, as shown in Figure 2b. In the first cycle scaled with increasing voltages (Process I, Figure 2a), a peak at 224 cm^−1^ was observed over 1.11–1.21 V, belonging to the Fe─O bond, which is the same as the Raman mode of Fe hydroxides.^[^ ^46^, ^47^ ^]^ When the applied potential reached 1.31 V, a well‐defined peak appeared at 442 and 509 cm^−1^, which can be assigned to the vibration of Ni(II)−O in Ni(OH)_2_.^[^ ^48^, ^49^ ^]^ This vibration has been reported in phosphates and selenides and regarded as a transition phase of MOH before forming MOOH.^[^ ^19^, ^50^ ^]^ Simultaneously, the intensity of the bands for C─O─C, C─C, and C═O started to decrease, indicating the transition from oxalate to hydroxides or even oxyhydroxides with the oxidation occurred during 1.21‐1.31 V.^[^ ^19^, ^51^, ^52^, ^53^ ^]^ Therefore, we can understand that the Ni_0.6_Fe_0.4_C_2_O_4_ phase starts to undergo transition after 1.21 V and coexist with NiFe(OH)_x_ in the electrocatalyst at the potential of 1.31 V. Moreover, we presume that Fe species undergo more rapid reconstruction than Ni species, which will be further discussed in subsequent experiments. If the applied voltage increased to 1.41 V, all Raman bands related with oxalates (C─O─C, C─C, and C═O) disappeared, indicating the completely transform of the surface layer from Ni_0.6_Fe_0.4_C_2_O_4_. In addition, two sharp Raman vibrations appeared at 477 and 557 cm^−1^ after 1.31 V, which could be attributed to the bending vibration of the E_g_ band of Ni(III)−O and the stretching vibration of the A_1g_ band of Ni─O, respectively.^[^ ^19^, ^44^, ^45^, ^53^, ^54^ ^]^ The appearance of these two vibration bands indicates the formation of γ‐NiOOH at the OER potentials.^[^ ^23^, ^55^ ^]^ While we identified the formation of FeOOH in the XPS results, the Raman characteristics of FeOOH could not be observed during 1.31–1.41 V. The possible reason is that the Raman vibrations of NiOOH are too strong and mask the bands of FeOOH which have relatively low intensity.^[^ ^53^, ^56^, ^57^ ^]^ Based on the in situ Raman results examined over 1.11–1.41, we can interpret that the electrocatalyst experienced a surface reconstruction along the pathway of Ni_0.6_Fe_0.4_C_2_O_4_→NiFe(OH)_x_→R‐Ni_0.6_Fe_0.4_OOH. The final R‐Ni_0.6_Fe_0.4_OOH phase has been widely proven as the actual OER centers under oxidation potentials.^[^ ^55^, ^56^, ^58^, ^59^, ^60^ ^]^ With the further increase of potentials to 1.61 V, the intensity of all peaks was weakened, due to the generation of violent bubbles screened the signal detection.^[^ ^61^ ^]^

In Process II, with the applied voltages went backward the cathodic direction (II in Figure 2a), the two strong variations of γ‐NiOOH gradually decreased, while the signal associated with highly disordering Ni(OH)_2_^[^ ^51^, ^62^ ^]^ and FeOOH^[^ ^63^, ^64^ ^]^ appeared near 528 cm^−1^ (Figure S13, Supporting Information), indicating that γ‐NiOOH transformed back to the Ni(OH)_2_ phase. Meanwhile, two characteristic vibrations at 292 and 696 cm^−1^ were detected, which are ascribed to the vibrations of Fe(III)−O species, implying the existence of FeOOH in the CV discharge process.^[^ ^43^ ^]^ However, no Ni_0.6_Fe_0.4_C_2_O_4_ could be detected anymore, due to the irreversible oxidation of oxalates. As a result, R‐Ni_0.6_Fe_0.4_OOH was reduced to amorphous Ni(OH)_2_‐FeOOH composite phase in Process II. In Process III, with the potential re‐increased to 1.41 V (III in Figure 2a), two peaks reappeared at 477 and 557 cm^−1^, declaring the re‐formation of γ‐NiOOH. Compared with the processes of I and II, a blue‐shift ≈4 cm^−1^ of the stretching band Ni(III)−O located at 557cm^−1^ was found in Process III, as presented in the Raman spectra collected at 1.41 V (Figure 2c), which means the shortening of the Ni(III)─O bond length in γ‐NiOOH.^[^ ^45^ ^]^ We also calculated the intensity ratio (*I*_477_/*I*_557_) of Ni(III)−O peaks at 1.41 V, where the intensity ratios decreased with the proceeding of cycling from Processes I to II, and then III. (Figure 2c), suggesting the upsurge of the disordering of the lattice. Further prolonging the cycles to 30 (XXX), 40 (XL), 50 (L), and 608 (LX) runs (Figure 2c; Figure S14, Supporting Information), the reconstructed surface eventually evolved into amorphous,^[^ ^43^, ^45^, ^53^ ^]^ and the two distinct peaks of NiOOH (400–600 cm^−1^) no longer appeared but only a very broad and unobvious bump. Therefore, based on the above results, the surface reconstruction process can be summarized as shown in Figure 2d. Upon applied potentials, Ni_0.6_Fe_0.4_C_2_O_4_ is first oxidized into NiFe(OH)_x_ after 1.21 V and then further oxidized into R‐Ni_0.6_Fe_0.4_OOH at higher oxidation potentials (Process I). During the discharging course (Process II), NiOOH in R‐Ni_0.6_Fe_0.4_OOH(I) transforms to a Ni(OH)_2_ phase and makes the R‐Ni_0.6_Fe_0.4_OOH(I) surface structure change into a Ni(OH)_2_‐FeOOH(II) composite structure. Further performing the cycling (Process III), R‐Ni_0.6_Fe_0.4_OOH(III) surface phased re‐forms, verifying the reversible transition between the Ni(OH)_2_‐FeOOH phase and R‐Ni_0.6_Fe_0.4_OOH but with intensified lattice disordering. Continuously proceeding the cycles to 30 rounds, the surface is completely reconstructed into amorphous R‐Ni_0.6_Fe_0.4_OOH.

**Figure 3:**
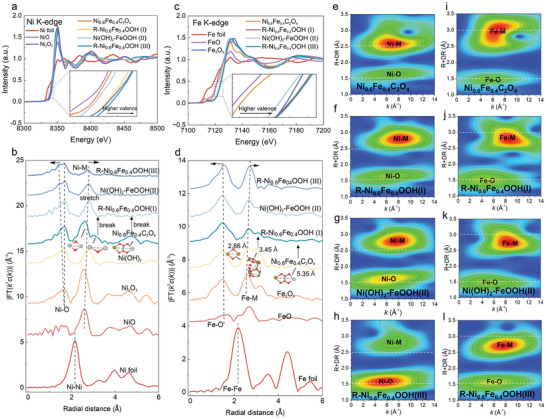
Quasi‐in situ XANES/EXAFS characterization monitoring on surface reconstruction. a) Ni K‐edge XANES collected at different electrochemical processes. b) Corresponding Ni k^3^‐weighted Fourier‐transformed EXAFS R‐space patterns in reconstruction. c) Fe K‐edge XANES collected at different reconstruction stages. d) Corresponding Fe k^3^‐weighted Fourier‐transformed EXAFS R‐space patterns. e) Ni K‐edge EXAFS oscillation functions k^2^χ(k) and k^2^‐weighted WT plots of Ni_0.6_Fe_0.4_C_2_O_4_. f) Ni K‐edge EXAFS oscillation functions k^2^χ(k) and k^2^‐weighted WT plots of R‐Ni_0.6_Fe_0.4_OOH(g) Ni K‐edge EXAFS oscillation functions k^2^χ(k) and k^2^‐weighted WT plots of Ni(OH)_2_‐FeOOH(II). h) Ni K‐edge EXAFS oscillation functions k^2^χ(k) and k^2^‐weighted WT plots of R‐Ni_0.6_Fe_0.4_OOH(III). i–l) Corresponding Fe K‐edge EXAFS oscillation functions k^2^χ(k) and k^2^‐weighted WT plots.

To disclose the electronic structure and local coordination environment, the Ni and Fe K‐edge X‐ray absorption near‐edge structure (XANES) and extended X‐ray absorption fine structure (EXAFS) spectroscopy characterizations of Ni_0.6_Fe_0.4_C_2_O_4_, R‐Ni_0.6_Fe_0.4_OOH(I), Ni(OH)_2_‐FeOOH(II), and R‐Ni_0.6_Fe_0.4_OOH(III) were performed under *quasi‐*in situ conditions along with standard reference substrates. As shown in the Ni K‐edge spectra in **Figure** **3**a, the relevant absorption edges of Ni_0.6_Fe_0.4_C_2_O_4_, NiO, and Ni(OH)_2_‐FeOOH(II) occurred at very close positions, suggesting the existence of typical Ni^2+^ species in Ni_0.6_Fe_0.4_C_2_O_4_ and Ni(OH)_2_‐FeOOH(II). A right drifting of the absorption edges with the progresses from R‐Ni_0.6_Fe_0.4_OOH(I) to R‐Ni_0.6_Fe_0.4_OOH(III) was observed, which are also located at a higher energy than those of Ni_0.6_Fe_0.4_C_2_O_4_, NiO, and Ni(OH)_2_‐FeOOH(II), proving the appearance of Ni^3+^ species in the reconstructed surface. Specifically, the higher energy state of R‐Ni_0.6_Fe_0.4_OOH(III) to R‐Ni_0.6_Fe_0.4_OOH(I) and Ni_2_O_3_ implies a higher valence state (Ni^3+δ^, 0 < δ < 1) as well as a higher number of defects in the defective R‐Ni_0.6_Fe_0.4_OOH(III).^[^ ^65^ ^]^ The Fourier transform of the EXAFS signal provides a photoelectron scattering profile as a function of the radial distance. Figure 3b presents the configurations of Ni species resolved from the EXAFS. The Ni─O bond ≈1.7 Å in R‐Ni_0.6_Fe_0.4_OOH was shifted to a lower energy compared with Ni(OH)_2_, which means that the Ni^2+^ in Ni(OH)_2_ changed to the Ni^3+δ^ in NiOOH with a contracted Ni─O bond length.^[^ ^60^, ^65^, ^66^ ^]^ Notably, the average coordination number of the Ni─O in R‐Ni_0.6_Fe_0.4_OOH (Table S1, Supporting Information) is apparently smaller than that of Ni(OH)_2_‐FeOOH(II), demonstrating a more unsaturated coordination of the Ni metal sites in R‐Ni_0.6_Fe_0.4_OOH. A stronger correlation was found not only between Ni−O but also between the nearest Ni─M bonds. The Ni−M (M = Ni/Fe) local spacing of the R‐NiFeOOH(I) and R‐NiFeOOH(III) were much longer compared with those in Ni_2_O_3_, because that the rich vacancy defects lead to the relaxation of the neighboring Ni or Fe sites. Three Ni─M bonds in Ni_0.6_Fe_0.4_C_2_O_4_ with bonds lengths of 2.86, 3.45, and 5.35Å were identified. The intensity of the bonds located at 3.45 and 5.35Å were significantly reduced with the progresses of electrochemical reconstruction, which means the breaking of local Ni─O or Ni─C─O bonds.

For the Fe K‐edge spectra (Figure 3c), R‐Ni_0.6_Fe_0.4_OOH(I), Ni(OH)_2_‐FeOOH(II), R‐Ni_0.6_Fe_0.4_OOH(III), and Fe_2_O_3_ displayed negative absorption edge shifts relative to the initial Ni_0.6_Fe_0.4_C_2_O_4_ and the FeO reference. It reveals that the valence state of Fe increases in R‐Ni_0.6_Fe_0.4_OOH(I), Ni(OH)_2_‐FeOOH(II), and R‐Ni_0.6_Fe_0.4_OOH(III). The valence state of Fe in Ni(OH)_2_‐FeOOH(II) was between FeO and Fe_2_O_3_, which means that Fe^2+^ and Fe^3+^ coexist in FeOOH but are dominated by Fe^3+^. Similarly, to Ni species, the presence of Fe^3+δ^ in R‐Ni_0.6_Fe_0.4_OOH should be the real site for high‐efficiency electrocatalysis.^[^ ^67^, ^68^ ^]^ In the Fe K‐edge EXAFS (Figure 3d), two conspicuous peaks located at 1.5 and 2.6 Å were assigned to the bonds of Fe─O and Fe─M, respectively, based on the data fitting and bond length analyses shown in Table S2 (Supporting Information). As marked by the arrows in Figure 3d, the Fe─O bonds were shifted to lower radial distances, indicating shorter Fe─O bonds, whilst the Fe─M bonds were shifted to higher radial distances for longer Fe─M bonds with the processes of the cycles and the evolution of R‐Ni_0.6_Fe_0.4_OOH. The Ni_0.6_Fe_0.4_C_2_O_4_, R‐Ni_0.6_Fe_0.4_OOH(I), Ni(OH)_2_‐FeOOH(II), and R‐Ni_0.6_Fe_0.4_OOH(III) showed markedly different *k*‐space oscillations (Figures S15 and S16, Supporting Information), suggesting the altered local atomic environment with the application of voltages. The wavelet transforms (WT) for the *k* ^2^‐weighted Ni/Fe K‐edge EXAFS of Ni_0.6_Fe_0.4_C_2_O_4_, R‐Ni_0.6_Fe_0.4_OOH(I), Ni(OH)_2_‐FeOOH(II), and R‐Ni_0.6_Fe_0.4_OOH(III) (Figure 3e,f) were extracted by applying EXAFS curve‐fitting routines, which further validate the structural transformation from Ni_0.6_Fe_0.4_C_2_O_4_ to R‐Ni_0.6_Fe_0.4_OOH. From the WT images of the *k*_3_‐weighted EXAFS, we could confirm the presences of both Ni/Fe‐M and Ni/Fe─O bonds at the beginning. Under reconstruction, the Ni─M bond lengths (Figure 3e–h) significantly relaxed, while the Ni─O bonds were slightly contracted, compared with the white dash lines. The anisotropic lattice expansions and contractions induce the disordering of the surface structure. In the contour diagram of Fe‐M (Figure 3i–l), it can be clearly seen that two kinds of bond lengths, 2.86 and 3.45 Å, of Fe‐M existed. After reconstruction, Fe─M bonds with a longer length (3.45 Å) disappeared first, leaving after the shorter Fe─M bond (2.86 Å) at a relatively stable state. This also explains why the Ni_x_Fe_1‐x_C_2_O_4_ with higher Fe contents were more unstable upon electrochemical processes.

### 2.3 Electrochemical Catalytic Activity of As‐Prepared and Reconstructed Catalysts

**Figure 4:**
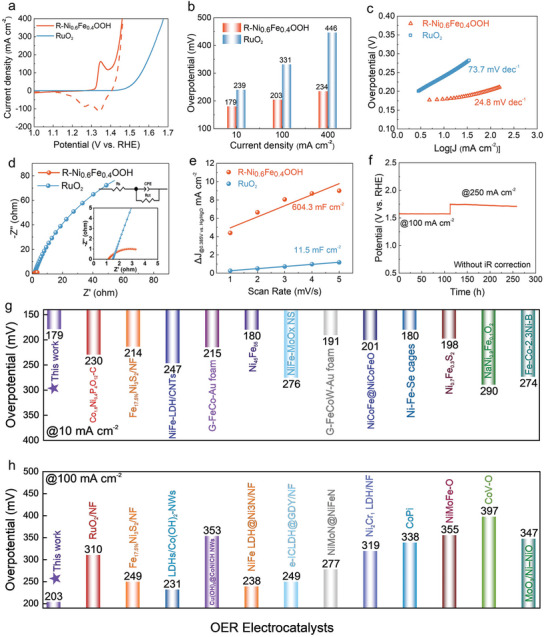
Electrochemical performance of reconstructed R‐Ni_0.6_Fe_0.4_OOH for OER catalysis. a) LSV curves of R‐Ni_0.6_Fe_0.4_OOH and RuO_2_ for OER, the dashed lines are the result of negative potential scanning. b) Comparison of overpotentials to reach 10, 100, and 400 mA cm^−2^. c) Tafel plots in OER catalysis. d) EIS plots of R‐Ni_0.6_Fe_0.4_OOH and RuO_2_ catalysts, the inset shows the equivalent circuit for EIS fitting. e) ECSA estimated from *C*_dl_ values. f) Chronopotentiometric curve of R‐Ni_0.6_Fe_0.4_OOH for OER at a current density of 10 cm^−2^ for 250 h. g) Overpotentials to reach 10 mV cm^−2^ of R‐Ni_0.6_Fe_0.4_OOH and some reported transition metal‐based OER catalysts. h) Comparison of overpotentials to reach 100 mV cm^−2^.

The electrochemical performances of the reconstructed electrocatalysts for OER catalysis were evaluated by a standard three‐electrode configuration in a 1 m KOH solution at 25 °C. As the linear sweep voltammetry curves (LSV) shown in Figure S17 (Supporting Information), the OER electrochemical activity of the reconstructed‐Ni_0.6_Fe_0.4_OOH was 21 times higher than that of the original‐Ni_0.6_Fe_0.4_C_2_O_4_ at an overpotential of 1.6 V. **Figure** **4**a shows the LSV of the fully reconstructed R‐Ni_0.6_Fe_0.4_OOH, which was obtained after 50 CV cycles, in comparison with the commercial RuO_2_. The results indicate that R‐Ni_0.6_Fe_0.4_OOH performed much higher activity than the commercial RuO_2_. Ultralow overpotentials of 179, 203, and 234 mV, respectively, were needed to reach current densities of 10, 100, and 400 mA cm^−2^, while 239, 331, and 446 mV, respectively, were requested for the commercial RuO_2_ (Figure 4b). Notably, two cathodic peaks at the potentials of 1.25 and 1.35 V in the LSV curve of Figure 4a can be attributed to the reduction of γ‐NiOOH to β‐NiOOH and then to Ni(OH)_2_, respectively. Typically, γ‐NiOOH contains more high valence Ni species (Ni^4+^, Ni^3+^) and is therefore first reduced to β‐NiOOH (Ni^3+^) at 1.35 V, and then to Ni(OH)_2_ with the decrease of the applied potentials, which confirms that γ‐NiOOH serves as the OER active species.^[^ ^17^, ^56^, ^69^ ^]^ The fitted Tafel plots (Figure 4c) and unfitted Tafel plots (Figure S18, Supporting Information) showed an extremely low Tafel slope of 24.8 mV dec^−1^ for R‐Ni_0.6_Fe_0.4_OOH, but it was 73.7 mV dec^−1^forRuO_2_, indicating a superior OER activity and more favorable dynamic characteristics of R‐Ni_0.6_Fe_0.4_OOH. In addition, a smaller *R*_ct_ of R‐Ni_0.6_Fe_0.4_OOH than RuO_2_ suggests a preferable charge transport ability of the R‐Ni_0.6_Fe_0.4_OOH (Figure 4d).^[^ ^70^ ^]^ The superior R‐Ni_0.6_Fe_0.4_OOH performance to RuO_2_ should be contributed by its more abundant active sites as indicated by its higher *C*_dl_ value (604.3 mF cm^−2^) than that of RuO_2_ (11.5 mF cm^−2^) (Figure 4e; Figure S19, Supporting Information).^[^ ^19^ ^]^

To provide a better figure on the OER catalytic activity of the reconstructed R‐Ni_0.6_Fe_0.4_OOH, we compared its overpotentials at 10 mA cm^−2^ (Figure 4g) and 100 mA cm^−2^ (Figure 4h) with the dominant transition metal‐based electrocatalysts. It is very clear that the reconstructed R‐Ni_0.6_Fe_0.4_OOH outperforms majority of the reported transition metal‐based electrocatalysts in OER catalysis. The long‐term electrochemical stability of R‐Ni_0.6_Fe_0.4_OOH was examined by using chronopotentiometric measurements at variable current densities without iR correction. As shown in Figure 4f, no significant decrease in activity at high current densities of 100 and 250 mA cm^−2^ was observed for more than 250 h tests. The LSV after long‐term stability tests also confirmed that R‐Ni_0.6_Fe_0.4_OOH still showed prominent activity after 250 h (Figure S20, Supporting Information). Based on the above evaluations, R‐Ni_0.6_Fe_0.4_OOH is a highly active and stable OER electrocatalyst with a practical application potential to operate at high current densities.

**Figure 5:**
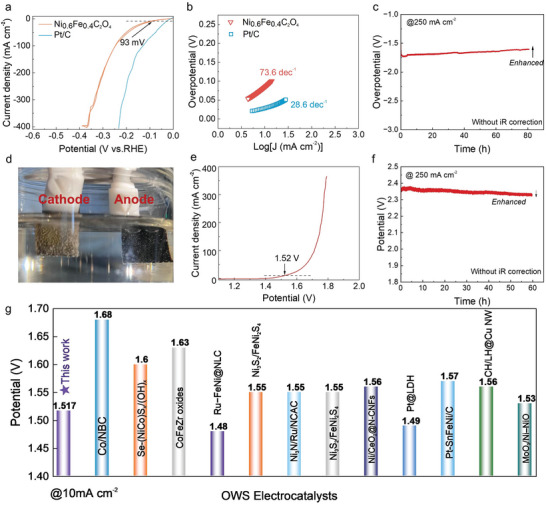
HER of as‐prepared Ni_0.6_Fe_0.4_C_2_O_4_ and overall water splitting performance of a Ni_0.6_Fe_0.4_C_2_O_4_|| R‐Ni_0.6_Fe_0.4_OOH electrolyzer. a) LSV curves of Ni_0.6_Fe_0.4_C_2_O_4_ and Pt/C for HER. b) Tafel plots of Ni_0.6_Fe_0.4_C_2_O_4_ and Pt/C. c) Chronopotentiometric curve of Ni_0.6_Fe_0.4_C_2_O_4_ for HER at a current density of 250 mA cm^−2^ for 60 h. d) Photograph of an overall water splitting (OWS) cell composed of a Ni_0.6_Fe_0.4_C_2_O_4_ cathode and a R‐Ni_0.6_Fe_0.4_OOH anode. e) LSV curves of OWS in 1 m KOH. f) Chronopotentiometry curve at a constant current density of 250 mA cm^−2^ for OWS. g) OWS Overpotentials of reported noble‐metal‐based and non‐noble‐metal‐based electrocatalysts at 10 mA cm^−2^.

The HER catalytic activity of the as‐prepared Ni_0.6_Fe_0.4_C_2_O_4_ was examined in a 1 m KOH solution at 25 °C. **Figure** **5**a shows the HER LSV curves of Ni_0.6_Fe_0.4_C_2_O_4_ together with the commercial Pt/C. Ni_0.6_Fe_0.4_C_2_O_4_ exhibits an overpotential of 93 mV to reach 10 cm^−2^ with a Tafel slope of 73.6 dec^−1^, which are a bit higher than 29 mV and 28.6 dec^−1^, respectively, for Pt/C. Both the XRD and in situ Raman characterizations confirmed that Ni_0.6_Fe_0.4_C_2_O_4_ remains stable without undergoing surface reconstruction throughout the HER process (Figure S21, Supporting Information). Even though an overall inferior HER activity of Ni_0.6_Fe_0.4_C_2_O_4_ to Pt/C resulted by a slightly higher mass transport resistance (Figure S22, Supporting Information) and lower ECSA value (Figures S23 and S24, Supporting Information), the intrinsic activity of Ni_0.6_Fe_0.4_C_2_O_4_ evaluated by normalizing ECSA by LSV (LSV_ECSA_) is higher than that of Pt/C (Figure S25, Supporting Information), suggesting faster reaction at each active site and higher intrinsic activity of Ni_0.6_Fe_0.4_C_2_O_4_ in this alkalic system.^[^ ^71^ ^]^ Furthermore, Ni_0.6_Fe_0.4_C_2_O_4_ has excellent HER stability, which maintained mostly stable overpotential at 250 mA cm^−2^ for more than 65 h (Figure 5c).

The excellent OER activity presented by R‐Ni_0.6_Fe_0.4_OOH and HER performance by the as‐synthesized Ni_0.6_Fe_0.4_C_2_O_4_ provide potential bifunctionality to construct an alkaline water electrolyzer with a configuration of Ni_0.6_Fe_0.4_C_2_O_4_||R‐Ni_0.6_Fe_0.4_OOH (Figure 5d), where the cathode loaded with Ni_0.6_Fe_0.4_C_2_O_4_ retained at its original yellow‐green color and the anode with R‐Ni_0.6_Fe_0.4_OOH was in black colour. As shown in the photograph in Figure 5d, violent bubbles generated from both electrodes. As shown in Figure 5e, a cell voltage of 1.52 V is needed to reach a current density of 10 mA cm^−2^. Besides the ultralow overpotential at low voltages, this bifunctional catalyst also presented a very promising potential for overall water splitting at large current densities. It only needed 1.71 V for reaching 100 mA cm^−2^, 1.75 V for 200 mA cm^−2^, and 1.77 V for 300 mA cm^−2^. It is very impressive that the water splitting electrolyzer proves superior stability for over 60 h, as demonstrated by the chronopotentiometry test performed at 250 mA cm^−2^ (Figure 5f). Even though the cathode presents a bit lower catalytic performance than that of commercial Pt/C, the outstanding anode performance well compensates the overall water splitting reaction performance. As shown in Figure 5g, the bifunctionality resulted by the in situ conversion of the NiFe oxalates into NiFe oxyhydroxides offers outstanding water splitting catalytic activity, which can match with the performance of some noble‐metal‐based catalysts and much outperforms the transition metal (oxide)‐based electrolyzers.^[^ ^22^, ^72^ ^]^ The obtained Ni_0.6_Fe_0.4_C_2_O_4_ and the converted catalysts have the characteristics of easy synthesis, homogeneous product, and reproducible large volume production, together with excellent catalytic activity and long‐term stability, which endow a strong potential for practical applications.

### 2.4 Roles of Fe in Surface Reconstruction and Electrochemical Performance

**Figure 6:**
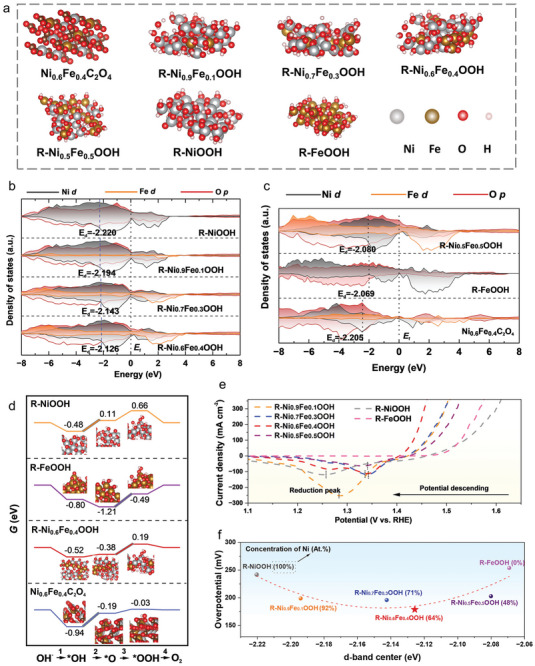
Effect of Fe content on catalytic activity of R‐Ni_x_Fe_1‐x_OOH. a) Structure of reconstructed amorphous R‐NiOOH, R‐Ni_x_Fe_1‐x_OOH, R‐FeOOH, and crystalline Ni_0.6_Fe_0.4_C_2_O_4_. b,c) Calculated DOS of amorphous R‐NiOOH, R‐Ni_x_Fe_1‐x_OOH, R‐FeOOH, and crystalline Ni_0.6_Fe_0.4_C_2_O_4_. d) Calculated free‐energy (eV) profile of OER processes on amorphous R‐NiOOH, R‐Ni_0.6_Fe_0.4_OOH, R‐FeOOH, and crystalline Ni_0.6_Fe_0.4_C_2_O_4_ (U = 1.23 V). e) OER‐LSV curves of R‐NiOOH, R‐Ni_x_Fe_1‐x_OOH, R‐FeOOH, and for OER. f) Volcano relationship between the calculated d‐band centers and the measured overpotentials at 10 mA cm^−2^ of R‐Ni_x_Fe_1‐x_OOH.

The effect of Ni/Fe content in Ni_0.6_Fe_0.4_C_2_O_4_ (*x* = 1, 0.9, 0.7, 0.6, 0.5, and 0) on both the surface reconstruction kinetics and electrochemical performance were examined. During the surface reconstruction induced by electrochemical cycling, besides the distinct changes of the colors of Ni_0.6_Fe_0.4_C_2_O_4_ (Figure S4a, Supporting Information), XRD characterizations (Figure S4b–g, Supporting Information) demonstrate that FeC_2_O_4_ had the rapidest reconstruction (Figure S25g, Supporting Information), which was fully completed after 10 CV cycles, while NiC_2_O_4_ had the slowest reconstruction which yet maintained obvious oxalate phase even after 50 CV cycles (Figure S4b, Supporting Information). The corresponding CV curves collected at different cycles on various Ni_x_Fe_1‐x_C_2_O_4_ samples confirmed that these with higher Fe contents had more favorable reconstruction kinetics (Figure S4h–m, Supporting Information), where lower current densities were needed to complete their phase transition. In addition, with the increase of Fe content, the reduction peak of Ni_0.6_Fe_0.4_C_2_O_4_ moved to a higher potential, confirming the critical role of Fe on the reconstruction (**Figure** **6**e).^[^ ^58^, ^66^ ^]^ Therefore, the Fe content plays a key role in modulating the surface reconstruction kinetics.

The Ni/Fe content also plays a critical role in the catalytic activity of the NiFe oxalates and oxyhydroxides. The reconstructed oxyhydroxide electrocatalysts of R‐NiOOH, R‐Ni_0.9_Fe_0.1_OOH, R‐Ni_0.7_Fe_0.3_OOH, R‐Ni_0.6_Fe_0.4_OOH, R‐Ni_0.5_Fe_0.5_OOH, and R‐FeOOH were obtained from the corresponding oxalates, as the crystal structures shown in Figure 6a. The R‐Ni_0.6_Fe_0.4_OOH exhibited the best OER performance with an overpotential of 179 mV at 10 mA cm^−2^, which far exceeds those of R‐NiOOH (242 mV), R‐FeOOH (254 mV), and R‐Ni_x_Fe_1‐x_OOH with other *x* values. This suggests that Fe partially replacing Ni atoms in NiOOH can significantly improve the OER performance of R‐NiOOH. Moreover, the EIS measurements exhibited an enhancement in conductivity with Fe addition, but this is inadequate to explain the changes in OER performance (Figure S26, Supporting Information).

To further explore the connections between the Fe content and the OER performance in the reconstructed R‐Ni_x_Fe_1‐x_OOH, density functional theory (DFT) calculations were conducted based on the phase structures shown in Figure 6a. It has been well reported that the OER activity is determined by the binding strength of active intermediates (_*_OH, _*_O, and _*_OOH) on the surface of electrocatalysts, as described in the d‐band center theory.^[^ ^73^, ^74^, ^75^, ^76^, ^77^ ^]^ As shown in Figure 6b,c, the density of states (DOS) of all catalyst surfaces close to *E*_f_ were principally derived from the Ni *d* and Fe *d* states. At the same time, the Fe substitution enhanced the *d*‐states near the *E*_f_ and enabled the *E*_d_ energy levels move toward *E*_f_, which profoundly affect the adsorption energy of intermediates on the R‐Ni_x_Fe_1‐x_OOH surfaces.^[^ ^75^, ^77^ ^]^ In detail, R‐NiOOH had the lowest *E*_d_ energy level, leading to a weak adsorption of the OER active species for proceeding an effective OER process. The Fe substitution raised the *E*_d_ energy level of R‐NiOOH and decreased the electron filling of antibonding states, and thus significantly facilitated the adsorption behavior of OER intermediates. Remarkably, a volcano relationship between the overpotentials and the calculated *E*_d_ energy levels rather than a linear relationship were identified, where the R‐Ni_0.6_Fe_0.4_OOH catalyst has the most favorable OER activity at a mediate *E*_d_ value (Figure 6e,f). The reason is that the higher *E*_d_ values at higher Fe contents result in over strong bonding energy, which resists the desorption behavior of intermediates and O_2_ release, and ultimately leads to decreased OER activity.^[^ ^78^ ^]^ Figure 6d presents the calculated Gibbs free energy of _*_OH, _*_O, and _*_OOH species on R‐NiOOH, R‐FeOOH, Ni_0.6_Fe_0.4_C_2_O_4_, and R‐ Ni_0.6_Fe_0.4_OOH. The step of _*_OH → _*_O is the rate‐determining step (RDS) with an energy barrier of 0.59 eV in R‐NiOOH. On R‐FeOOH and R‐ Ni_0.6_Fe_0.4_OOH, the step of _*_O → _*_OOH becomes the RDS with energy barriers of 0.72 and 0.19 eV, respectively. Both the experimental results and theoretical calculations provide affirmative evidence that a proper Fe substitution can modulate the OER activity of R‐Ni_x_Fe_1‐x_OOH to reach an optimizing *d*‐band center for favorable intermediate adsorption.

Based on the quasi‐in situ and in situ structural characterizations, the theoretical calculations, and the catalytic activity evaluations, the relationship of structure‐component‐activity on the reconstructed NiFiOOH can be extracted. The XANES results (Figure 3a,c) show that both Ni and Fe in the R‐NiFeOOH exist in the form of M^(3+δ)^, high oxidation states. With the increase of Fe contents, the DOS (Figure 6b,c) indicates that the Fe states enhance the *d*‐band of Ni_x_Fe_1‐x_OOH to gradually move toward the Fermi level. These results indicate that the metal Ni atoms have a high valence configuration and non‐uniform charge distribution with Fe‐doping, and thus increased *d* band center and enhanced the adsorption capacity toward OER intermediates.^[^ ^22^, ^57^ ^]^ The free energy profiles (Figure 6d) also confirm that the R‐Ni_0.6_Fe_0.4_OOH has optimal adsorption of oxygen‐containing intermediates of_*_O, _*_OH, and _*_OOH. Instead of a linear relationship, a volcanic relationship between the OER overpotential and the *d*‐band center/Fe‐content for Ni_x_Fe_1‐x_OOH catalysts, as confirmed by the well‐known Sabatier principle and similar to previous studies on FiNi hydroxides,^[^ ^79^ ^]^ in which an appropriate *E*_d_ level must be present to balance the adsorption of the intermediates (_*_OH, _*_O, and _*_OOH) and the desorption of O_2_ (Figure 6d,f).^[^ ^3^, ^57^ ^]^

## 3 Conclusion

In summary, Ni_x_Fe_1‐x_C_2_O_4_ (*x* = 1, 0.9, 0.7, 0.6, 0.5, and 0) with controllable Ni/Fe ratio were prepared and the electrochemical structure reconstructions were investigated. By taking the advantages of in situ Raman spectroscopy and quasi‐in situ XANES/EXAFS characterizations, the surface structure reconstruction from the as‐prepared Ni_x_Fe_1‐x_C_2_O_4_ to defective Ni_x_Fe_1‐x_OOH and finally reconstructed amorphous Ni_x_Fe_1‐x_OOH (R‐Ni_x_Fe_1‐x_OOH) and the reversible Ni(OH)_2_‐FeOOH(II)⇋R‐Ni_x_Fe_1‐x_OOH reaction upon the ascending and descending CV scans and the proceeding cycles were identified. It is interesting that an underlying volcano relationship between the d‐band centers and the OER activity correlated with the Ni/Fe ratios was elucidated. Benefiting from the abundant active centers and the intermediate reactant adsorption‐desorption behavior, the fully reconstructed R‐Ni_0.6_Fe_0.4_OOH exhibited the optimal OER activity with an overpotential of 179 mV at 10 mA cm^−2^ and an ultra‐low Tafel slope of 24.8 mV dec^−1^ in 1 m KOH. Assembled the reconstructed R‐Ni_0.6_Fe_0.4_OOH with the as‐synthesized Ni_0.6_Fe_0.4_C_2_O_4_ possessing excellent HER performance into an alkaline water electrolyzer, an overall water splitting reached the current density of 10 mA cm^−2^ at a voltage of 1.52 V and maintained a stable operation for more than 120 h. Impressively, this overall splitting cell can reach a current density as high as 300 mA cm^−2^ at an overpotential of 1.77 V and maintain stable at 250 mA cm^−2^ for over 60 h, demonstrating its capability for practical applications. This work therefore not only provides an in‐depth mechanism understand on the in situ reconstruction of OER electrocatalysts but also offers an innovative to construct effective electrocatalysts for overall water splitting via in situ electrochemical reconstruction.

## Conflict of Interest

The authors declare no conflict of interest.

## Author Contributions

Z.Z. and X.Y.R. contributed equally to this work. All authors were involved into acquisition, analysis, and discussion and were given approval for the final version of the manuscript.

## Supporting information



Supporting Information

## Data Availability

The data that support the findings of this study are available from the corresponding author upon reasonable request.
