# Supplementary material for: In Situ Reconstructing NiFe Oxalate Toward Overall Water Splitting
Source: Adv Sci (Weinh). 2024 Oct 3;11(44):2408754. doi: 10.1002/advs.202408754 (PMC11600197; doi:10.1002/advs.202408754)
Supplement: Supplementary file 1 — Supporting Information [file ADVS-11-2408754-s001.docx]

Supporting Information

**In-situ Reconstructing NiFe oxalate toward Overall Water Splitting**

*Zhen Zhang^a^, Xiaoyu Ren^a^, Wenyuan Dai^a^, Hang Zhang^a^, Zhengyin Sun^a^, Zhuang Ye^a^, Ying Hou^a^, Peizhi Liu^a^, Bingshe Xu^a, b^, Lihua Qian^c^, Ting Liao^d, f^, Haixia Zhang^a^*, Junjie Guo^a^*, Ziqi Sun^e, f^**

*^a^*Key Laboratory of Interface Science and Engineering in Advanced Materials, Ministry of Education, College of Materials Science and Engineering, Taiyuan University of Technology, Taiyuan 030024, P. R. China

E-mail address: zhanghaixia@tyut.edu.cn; guojunjie@tyut.edu.cn

*^b^*Materials Institute of Atomic and Molecular Science, Shaanxi University of Science &Technology, Xi'an 710021, P. R. China

*^c^*School of Physics, Huazhong University of Science and Technology, Wuhan 430074, P. R. China

*^d^*School of Mechanical Medical and Process Engineering, Queensland University of Technology, George Street, Brisbane, QLD 4000, Australia

*^e^*School of Chemistry and Physics, Queensland University of Technology, Brisbane, QLD 4000, Australia

*^f^*Centre for Materials Science, Queensland University of Technology, 2 George Street, Brisbane 4000, Australia

E-mail: ziqi.sun@qut.edu.au

**Experimental sections**

*Chemicals and materials*

Lithium iron phosphate (LiFePO_4_) cathode powders was purchased from Macklin Ltd (Shanghai, China). Hydrochloric acid (HCl, AR.), ethanol (C_2_H_5_OH, AR.) and oxalic acid [(H_2_C_2_O_4_·2H_2_O), A.R] were purchased from Tianjin Chemical Work. Ammonium persulfate [(NH_4_)_2_S_2_O_8_, 98%] and Potassium hydroxide (KOH 98%) were purchased from Macklin Ltd (Shanghai, China). All the materials were utilized in the absence of further purification in this study. The deionized water employed in the whole experiments was prepared by an ultrapure purification system.

*LiFePO_4_ leaching*

Weighing 0.02 mol of LiFePO_4_, 0.04 mol of H_2_C_2_O_4_·2H_2_O and 0.01 mol (NH_4_)_2_S_2_O_8_ in a beaker, adding 250 mL of deionized water, the beaker was sealed and placed in a magnetic agitator at 25 °C for 24 h. Finally, the precursor solution of hydrothermal reaction was obtained by filtrating the black precipitation.

*Preparation of Ni_x_Fe_1-x_C_2_O_4_*

NF (1 cm × 3 cm) was cleaned under sonication in ethanol, 3 M HCl for 10 min, respectively. Ni_0.6_Fe_0.4_C_2_O_4_ was prepared through a one-step hydrothermal method. The 12 mL above-mentioned precursor solution was diluted in 24 mL water (a series of control samples were prepared by adjusting the ratio of precursors to deionized water, for Ni_0.9_Fe_0.1_C_2_O_4_, the 0.72 mL above-mentioned precursor solution was diluted in 35.28 mL water; for Ni_0.7_Fe_0.3_C_2_O_4_, the 3.6 mL above-mentioned precursor solution was diluted in 32.4 mL water; for Ni_0.5_Fe_0.5_C_2_O_4_, 36 mL above-mentioned precursor solution added into the teflon lining directly), continuing to add 0.121 g H_2_C_2_O_4_·2H_2_O and stirring until completely dissolved. The as-obtained solution together with one piece of cleaned NF (1 × 3 cm) was then sealed in a 50 mL Teflon-lined stainless-steel autoclave and heated at 180 °C for 12 h. The Ni^2+^ ions dissolved from NF and Fe^2+^ ions from LiFePO_4_ reacted with C_2_O_4_^2−^ to form homogeneous atomic-level blended Ni_x_Fe_1-x_C_2_O_4_ compounds. After being washed thoroughly with water, the as-prepared NF coated with Ni_x_Fe_1-x_C_2_O_4_ layer was dried at 60 °C.

*Preparation of NiC_2_O_4_*

Ni foams (1 cm × 3 cm, NF) were cleaned under sonication in ethanol, 3 M HCl for 10 min, respectively. Weighing 0.66 g H_2_C_2_O_4_·2H_2_O in a beaker with 36 mL of DI water and stirring until completely dissolved, the as-obtained solution together with one piece of cleaned NF (1 × 3 cm) were then sealed in a 50 mL Teflon-lined stainless-steel autoclave and heated at 180 °C for 12 h. After being washed thoroughly with water, the as-prepared NF coated with NiC_2_O_4_ layer was dried at 60 °C.

*Preparation of FeC_2_O_4_*

The 12 mL above-mentioned precursor solution was diluted in 24 mL water, continuing to add 0.121 g H_2_C_2_O_4_·2H_2_O and stirring until completely dissolved. The as-obtained solution was then sealed in a 50 mL Teflon-lined stainless-steel autoclave and heated at 180 °C for 12 h. The precipitate was collected, cleaned and dried at 60 °C in an oven for 12 h. The ink was obtained by dispersing Fe(C_2_O_4_)_2_ (100 mg) into a dispersant made up of 100 μL 5 wt% Nafion solution and 900 μL ethanol with the assistance of sonication for 30 min. The electrodes were obtained by dropping 500 μL ink onto washed NF and naturally dried at room temperature.

*Materials Characterization*

X-ray diffraction (XRD) measurements were carried out on a Rigaku Ultima IV (Cu Kα radiation). Morphology of the materials was observed using a SEM (TESCAN, LYRA3) and high-resolution transmission electron microscopy (HRTEM, JEOL, JEM-2010F). The energy dispersive X-ray (EDX) was collected using Oxford instruments equipped on SEM and TEM. X-ray photoelectron spectroscopy (XPS, Thermo Scientific ESCALAB Xi+) was performed to detect the bonding states of the materials. *In situ* Raman experiments were performed using a conjoint system of HORIBA HR EVO Raman devices (633 nm laser) and electrochemical workstation (CHI 760E). Quasi in situ XPS measurements were performed by taking the samples out of the electrolytic cell after different CVs (Figure 1g-i)). The Raman spectrogram was collected using Ni_0.6_Fe_0.4_C_2_O_4_ sample during the CVs at a scan rate of 1 mV s^-1^. Quasi in situ X-ray absorption near-edge structure (XANES) and EXAFS measurements were performed by taking the samples out of the electrolytic cell after process I, process II and process III (corresponding to In situ Raman in Figure 2a)). Ni, Fe K-edge X-ray absorption spectra (XAS) were collected at the SPring-8 BL14B2 using the transmission mode. Data reduction, data analysis, and extended X-ray absorption fine structure (EXAFS) fitting were performed and analyzed with the Athena and Artemis programs of the Demeter data analysis packages^[1]^ that utilizes the FEFF6 program^[2]^ to fit the EXAFS data. The energy calibration of the sample was conducted through a standard Ni foil, which as a reference was simultaneously measured. A linear function was subtracted from the pre-edge region, then the edge jump was normalized using Athena software. The χ(k) data were isolated by subtracting a smooth, third-order polynomial approximating the absorption background of an isolated atom. The k^3^-weighted χ(k) data were Fourier transformed after applying a Hanning window function. For EXAFS modeling, the global amplitude EXAFS (CN, R, σ^2^ and ΔE_0_) were obtained by nonlinear fitting, with least-squares refinement, of the EXAFS equation to the Fourier-transformed data in R-space, using Artemis software, EXAFS of the Fe foil is fitted and the obtained amplitude reduction factor S_0_^2^ value (0.82) was set in the EXAFS analysis to determine the coordination numbers (CNs) in the Ni-O and Ni-Ni scattering paths in the sample. For Wavelet Transform analysis,^[3]^ the χ(k) exported from Athena was imported into the Hama Fortran code. The parameters were listed as follow: R range, 1.0 – 6.0 Å, k range, 0–14 Å^-1^; k weight, 3; and Morlet function with κ=15, σ=1 was used as the mother wavelet to provide the overall distribution. X-ray photoelectron spectroscopy (XPS) characterizations were performed on a Thermo ESCALAB 250Xi X-ray photoelectron spectroscope using the home-made X-ray cell. At different applied potentials, the working electrodes were first stabilized to reach a steady state, then the corresponding XPS signals were collected and analyzed.

*Electrocatalytic Measurements*

Electrochemical measurements were performed in a standard three-electrode system on a CHI 760E electrochemical workstation with a Hg/HgO electrode as the reference electrode and a graphite rod as the counter electrode. All electrochemical measurements were carried out under 25 ^o^C in 1 M KOH (pH = 13.8). The measured potentials were transformed to reversible hydrogen electrode (RHE) on the basis of the equation E(RHE) = E(Hg/HgO) + 0.098 + 0.059 pH. The exposed geometrical area of electrode for electrochemical test was 1 × 1 cm^2^. Unless otherwise marked, all of the obtained linear sweeping voltammetry (LSV) curves were corrected via 90% iR auto-compensation. The polarization curves obtained from LSV measurements were recorded at a scan rate of 5 mV s^-1^. Before recording, the electrodes were operated at a scan rate of 100 mV s^−1^ until a stable cyclic voltammetry (CV) curve was obtained. The structure, composition and morphology of samples performing different CVs (0 CV, 3 CVs, 10 CVs, 20 CVs, 50 CVs…) at a scan rate of 100 mV s^-1^ were investigated by XPS, TEM, XRD. The long-term stability was tested using chronopotentiometric measurements at 100 mA cm^−2^ and 250 mA cm^-2^ without iR correction. The ECSA was estimated by the electrochemical *C*_dl_ from different scan rates (1-5 mV s^−1^) in a non-faradic region of 0.35 − 0.40 V and -0.875 − -0.7 (vs. Hg/HgO). The final obtained *C*_dl_ value was identical with half of the fitting linear slope. The ECSA-normalized current density (*J*_𝐸𝐶𝑆𝐴_) of OER electrocatalysts was calculated by *J*_𝐸𝐶𝑆𝐴_ = *J/ECSA*. The value of ECSA was estimated by the following equation ECSA= *C*_dl_ /C_s_, where the specific capacitance (C_s_) value is 0.04 mF cm^-2^ in 1 M KOH.^[4]^ All Electrochemical impedance spectrum (EIS) was gathered in a frequency range from 100 k to 0.01 Hz at 0.5 V vs. RHE.

*Density functional theory (DFT) calculations*

All DFT calculations were performed using Vienna ab initio simulation package (VASP)^[5]^ within the projector augmented-wave (PAW)^[6, 7]^ approach and the Perdew-Burke-Ernzerhof (PBE) generalized gradient approximation (GGA) + Hubbard U method.^[8, 9]^ The kinetic energy cut-off was set to 500 eV for the plane wave basis set. The atomic positions were fully optimized until all the residual forces on the atoms were less than 0.05 eV/Å, and the convergence threshold for self-consistent-field (SCF) iteration was set at 10^−6^ eV. A series of supercells (R-Ni_x_Fe_1-x_OOH, 15.2×13.7×19.5 Å; NiFeC_2_O_4_, 10.7×20.3×12.1 Å) were built for theoretical calculation and the vacuum layer of 15 Å was used to avoid the mirror interactions.

We fabricate the amorphous R-Ni_x_Fe_1-x_OOH structure by randomly arranging atoms in a virtual box, and then by breaking and rearranging the chemical bonds through continuous annealing and heating treatments. Atoms were first randomly thrown (Ni, Fe, O, H atoms) into a virtual cube box. Then, a thermodynamically stable structure was obtained by gradually increasing the temperature and cycling the same temperature several times with chemical bonds rearranged and broken continuously and stress relieved. The bond number and volume converge gradually to stable values as annealing and heating times are increased. The ideal bond value is determined by the product of the number of elements and the coordination number of elements except oxygen.

The four-electron mechanism is considered for OER, which includes four reaction steps:

*＋ H_2_O→*OH+ H+ + e^−^

*OH→*O+H^+^ +e^−^

*O+ H_2_O→*OOH^+^ H^+^ + e^−^

*OOH→O_2_＋H^+^ +e^−^

where * and X*represent an adsorption site and an adsorbed X intermediate on the surface, respectively. The free energy of H^+^+ e^−^ can be half of formation energy of H_2_ at 298 K and 1 atm. The free energy of the HER and OER is computed by the equation ΔG = ΔE+ ΔZPE − TΔS. The value of ΔE is obtained by the computation of geometrical structures. The values of ΔLPE and ΔS are determined by employing the computed vibrational frequencies and standard tables for the reactants and products in the gas phase. The entropy for the adsorbed atoms/molecules at the surface active site are assumed to be zero.


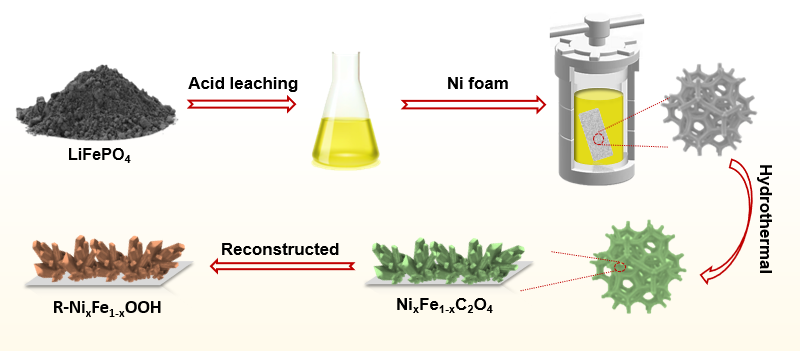


**Figure S1**. Schematic illustration of the synthesis of Ni_x_Fe_1-x_C_2_O_4_ and R-Ni_x_Fe_1-x_OOH.


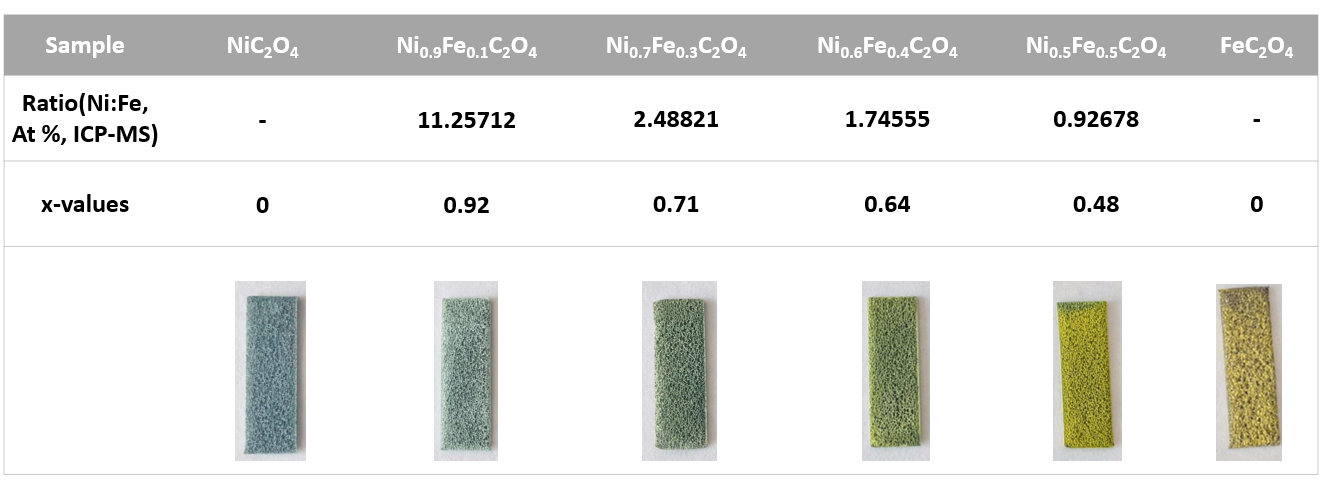


**Figure S2**. ICP-MS results and corresponding optical images of Ni_x_Fe_1-x_C_2_O_4_.

Optical photographs clearly show the change trend of the sample color from light blue to yellow as the Fe content increases. The color of the two ends of the sample is slightly different from that of the main body, which is because the substrate leans diagonally on the inner wall of the polytetrafluoroethylene during the hydrothermal growth, and the bottom of the substrate cannot be in good contact with the reaction solution.


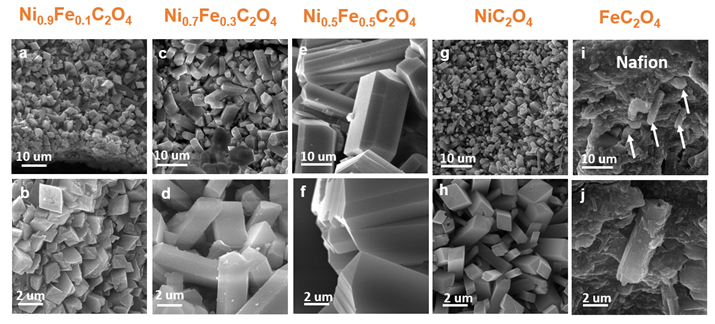


**Figure S3**. SEM images of (a, b) Ni_0.9_Fe_0.1_C_2_O_4_, (c, d) Ni_0.7_Fe_0.3_C_2_O_4_, (e, f) Ni_0.5_Fe_0.5_C_2_O_4_, (g, h) NiC_2_O_4_ and (i, j) FeC_2_O_4_.


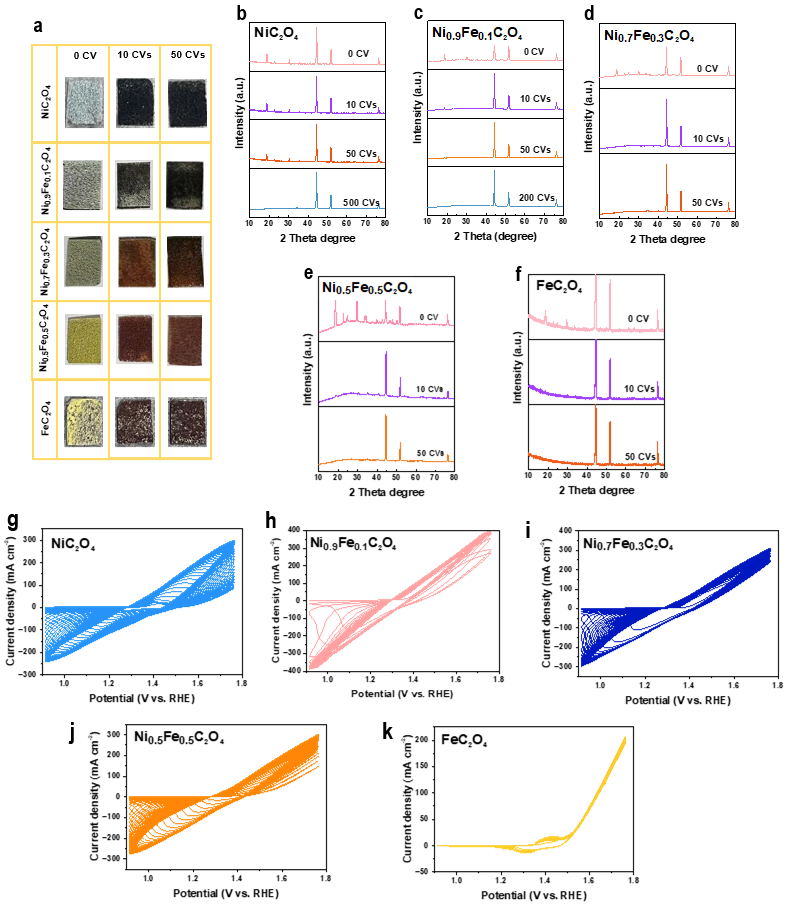


**Figure S4**. (a) Optical images of Ni_x_Fe_1-x_C_2_O_4_ after CVs (at a scan rate of 100 mV s^−1^) and corresponding (b-g) XRD patterns. (h-m) 50 CVs of Ni_x_Fe_1-x_C_2_O_4_ in 1 M KOH electrolyte at a scan rate of 100 mV s^−1^.

Under oxidation potential conditions during electrochemical tests, the catalyst is R-NiFeOOH, in which the black color of NiOOH blocks the yellowish-brown color of FeOOH, so that the overall color of the anode is close to black (**Figure 5d**). However, as we discussed in your previous comment, the NiOOH reduced back to Ni(OH)_2_ in air, which is in a color of light green. Combining with the primarily yellowish-brown color of FeOOH, a brown color appears on the post-tested samples. Of course, the reduction of NiOOH to Ni(OH)_2_ in air must take sufficient time. If we take the photos without long delay, we can still observe the original colors on the as-tested samples, as shown in **Figure S4a**.


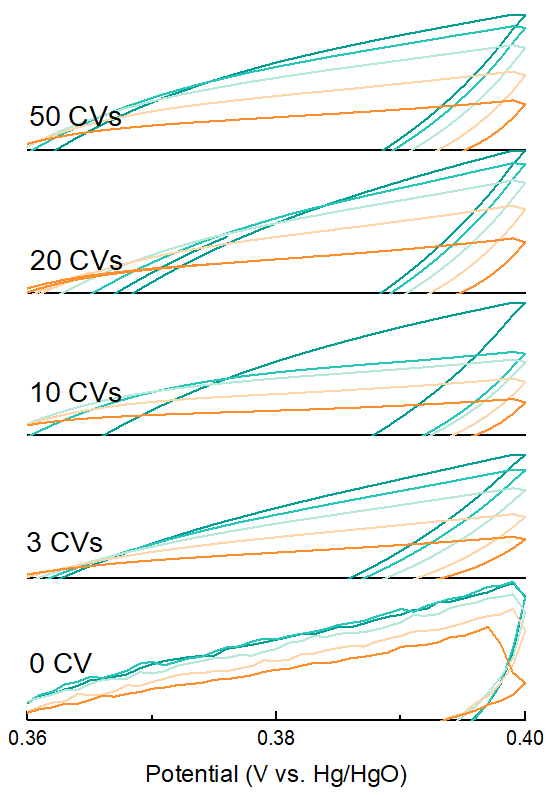


**Figure S5**. CV measurements at different scan rates. (a) Ni_0.6_Fe_0.4_C_2_O_4_, (b) Ni_0.6_Fe_0.4_C_2_O_4_ after 3 CVs reconstruction, (c) Ni_0.6_Fe_0.4_C_2_O_4_ after 10 CVs reconstruction, (d) Ni_0.6_Fe_0.4_C_2_O_4_ after 20 CVs reconstruction and (e) Ni_0.6_Fe_0.4_C_2_O_4_ after 50 CVs reconstruction in the potential range of 0.35–0.4 V vs. Hg/HgO at scan rates from 1 mV s^-1^ to 5 mV s^-1^.


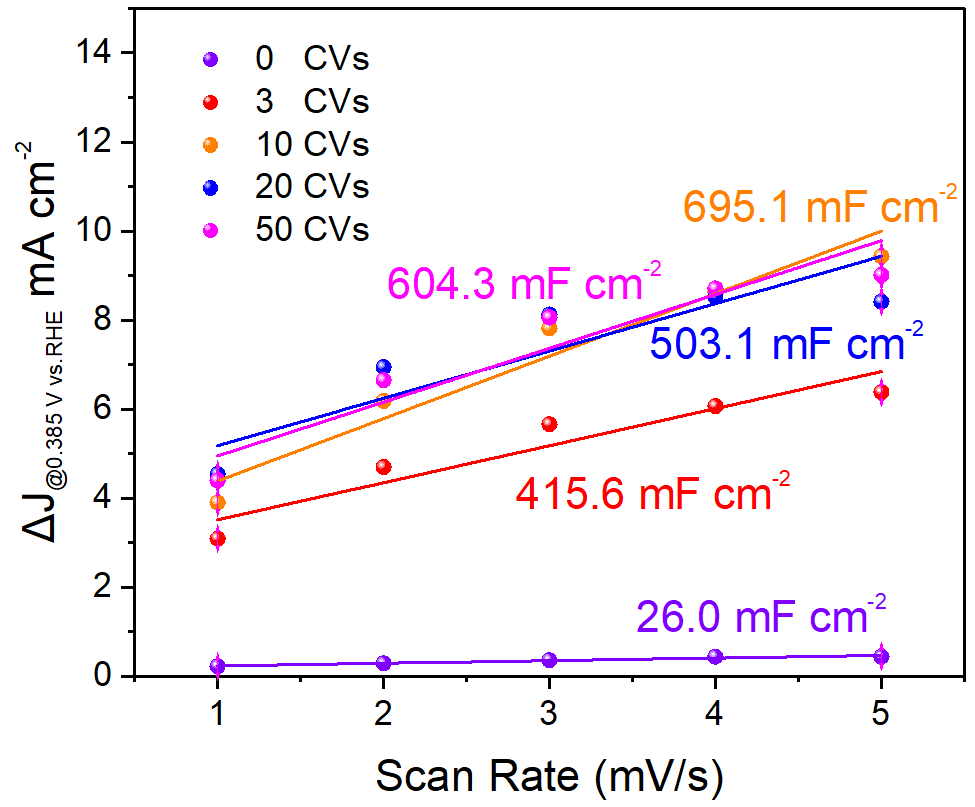


**Figure S6**. Capacitive currents at different scan rates for Ni_0.6_Fe_0.4_C_2_O_4_ after different CVs.


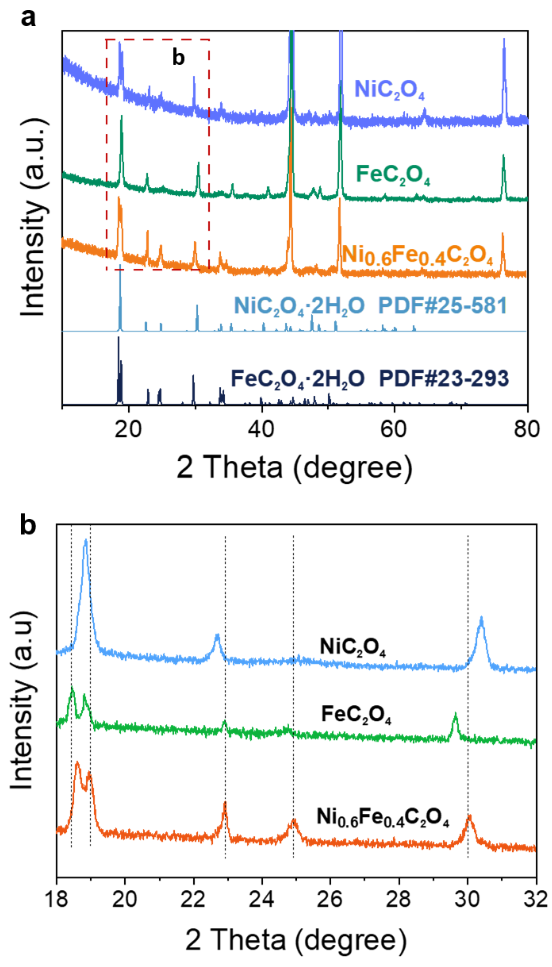


**Figure S7**. (a) XRD patterns of FeC_2_O_4_, NiC_2_O_4_ and Ni_0.6_Fe_0.4_C_2_O_4_. (b) Local zoom image of (a)

The different atomic radius of Ni (II) and Fe (II) leading to the changes of crystal plane spacing and thus resulting in the shift of XRD peaks.


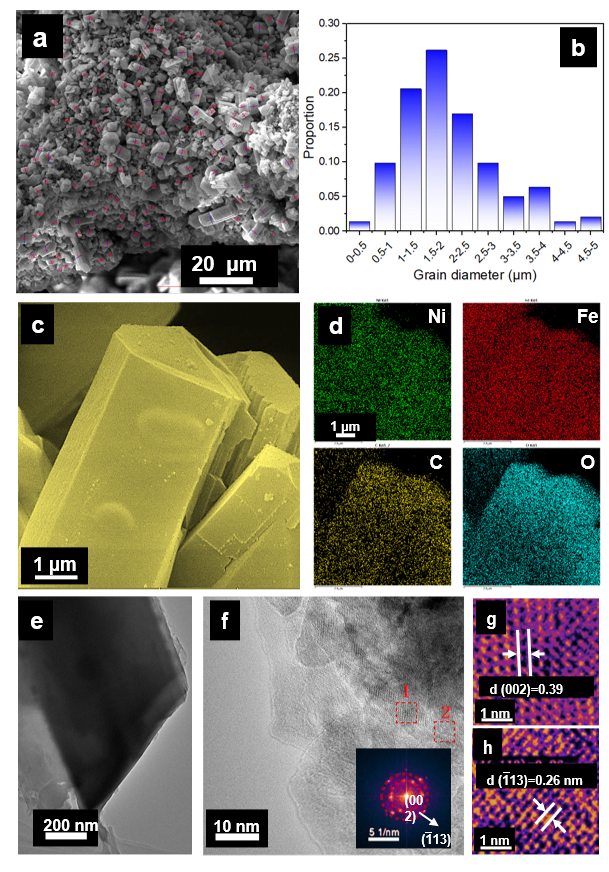


**Figure S8**. (a) SEM image of Ni_0.6_Fe_0.4_C_2_O_4_. (b) The corresponding particle size distribution map of Ni_0.6_Fe_0.4_C_2_O_4_. (c) SEM image of Ni_0.6_Fe_0.4_C_2_O_4_ and (d) the corresponding EDX mapping. (e) TEM and (F-H) HRTEM images of Ni_0.6_Fe_0.4_C_2_O_4_. Inset in (f) shows the fast fourier transform algorithm (FFT) pattern.


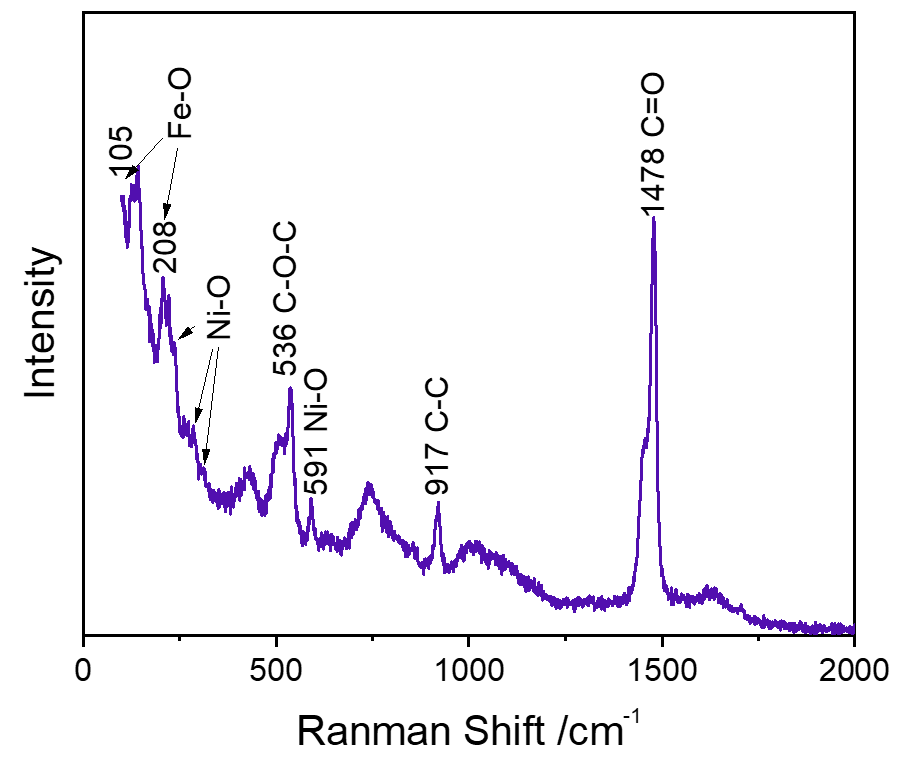


**Figure S9**. Raman spectrum of Ni_0.6_Fe_0.4_C_2_O_4_.


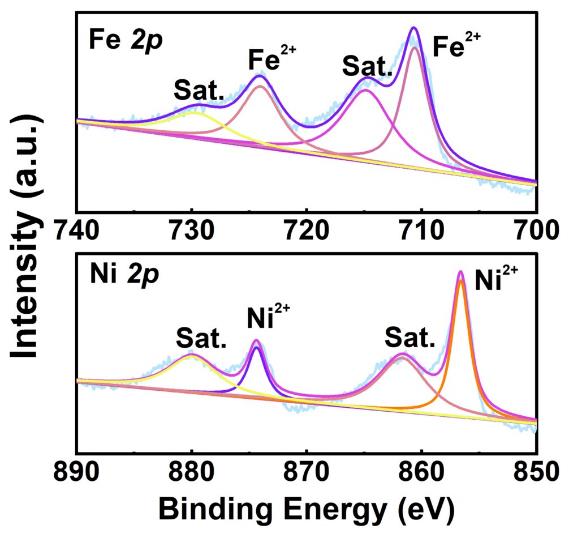


**Figure S10**. High-resolution XPS spectra of Ni and Fe in Ni_0.6_Fe_0.4_C_2_O_4_.


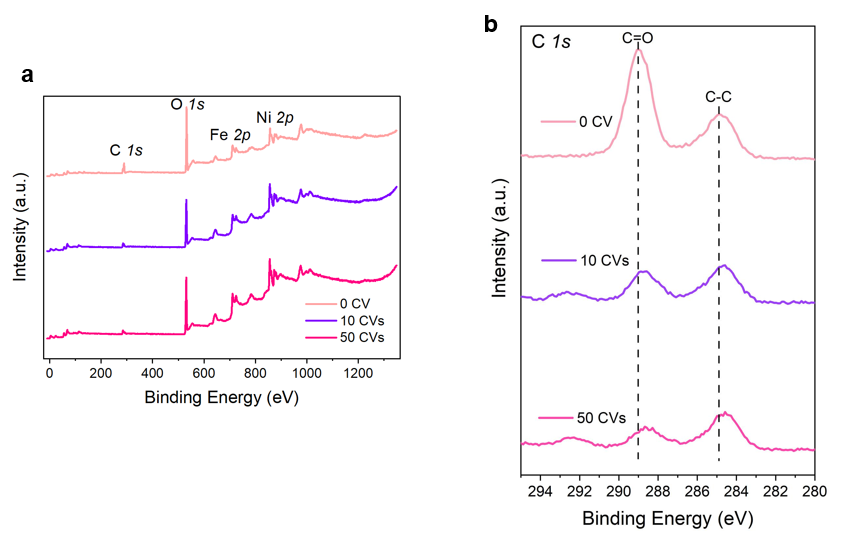


**Figure S11**. (a) XPS survey and (b) C 1s spectra of Ni_0.6_Fe_0.4_C_2_O_4_, Ni_0.6_Fe_0.4_C_2_O_4_ after 10 CVs and Ni_0.6_Fe_0.4_C_2_O_4_ after 50 CVs (at a scan rate of 100 mV s^−1^).


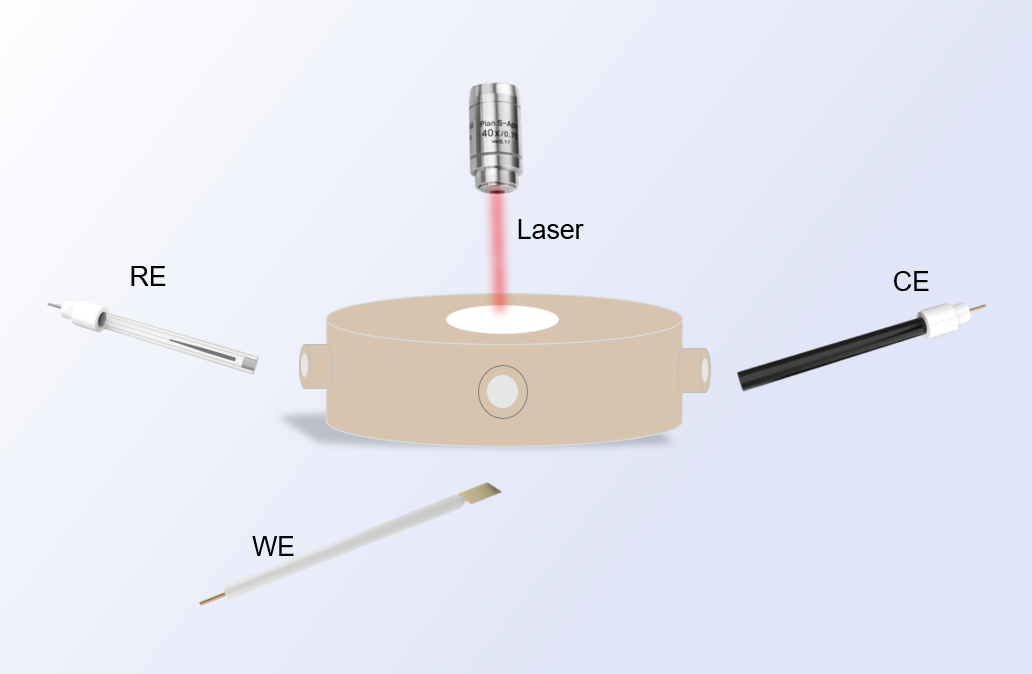


**Figure S12**. Simplified cell configuration of *in situ* Raman test.


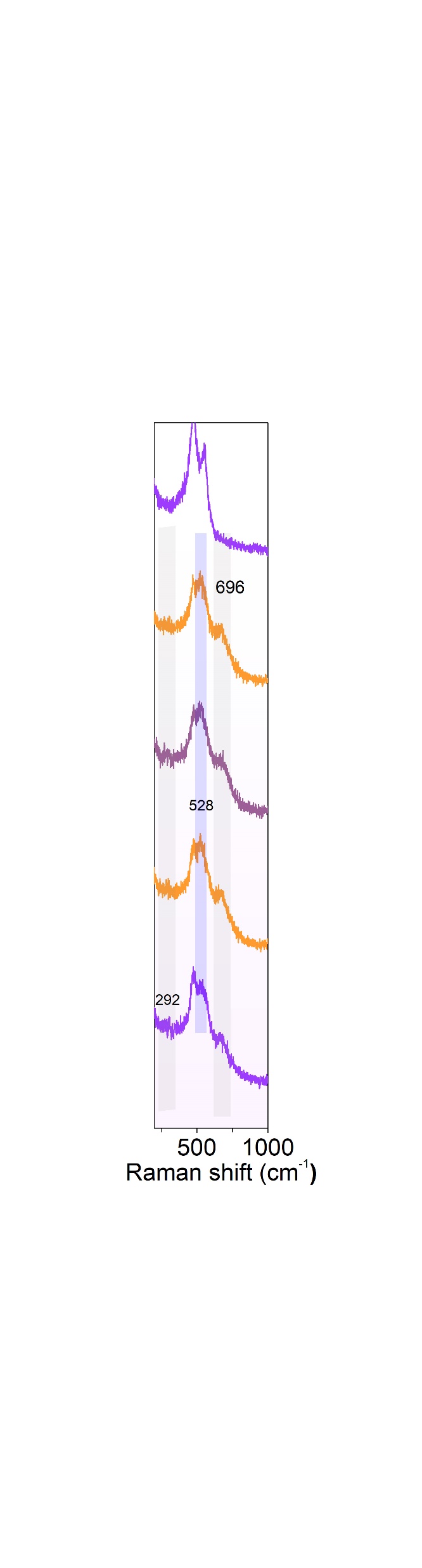


**Figure S13**. A partial enlargement of Figure 3A.


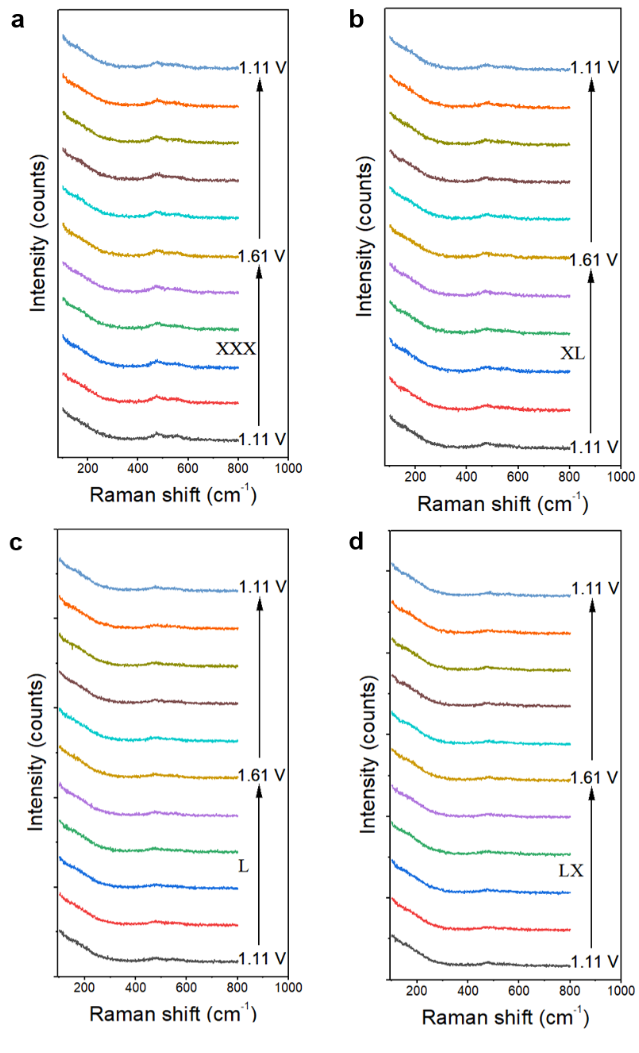


**Figure S14**. *In situ* Raman spectra captured for Ni_0.6_Fe_0.4_C_2_O_4_ electrocatalysts (XXX, XL, L and LX represents the thirtieth, fortieth, fiftieth and sixtieth process in the potential range of 1.11-1.61V, respectively) in 1 M KOH electrolyte at a scan rate of 1 mV s^−1^.


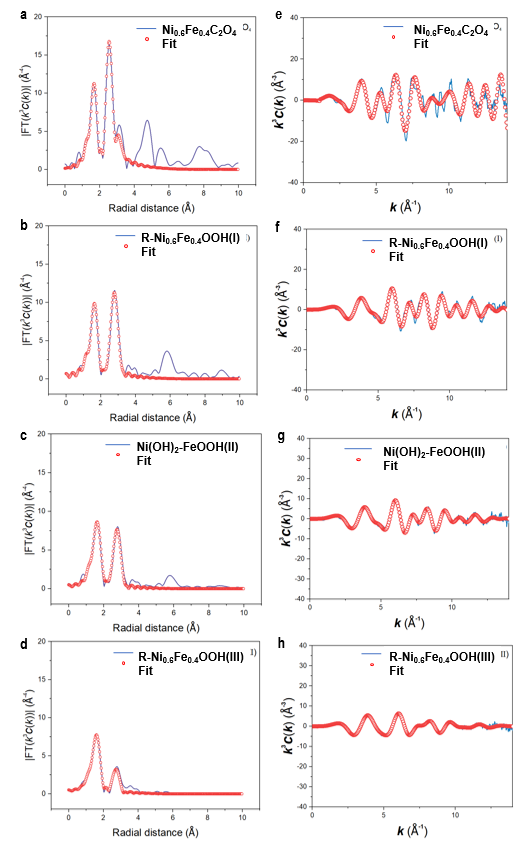


**Figure S15**. Fitting results of (a-h) Ni R/K-edge FT-EXAFS curves.

From the comparison of R space and this fitting results, it can be seen that the sample mainly contains Ni-O and Ni-Ni coordination. The coordination fitting between the sample and the model was carried out, and all fitting parameters were within a reasonable range. The specific fitting parameters are shown in **Table S1**.

For Wavelet Transform analysis, the χ(k) exported from Athena was imported into the Hama Fortran code.^[3]^ The parameters were listed as follow: R range, 1.0 - 6.0 Å, k range, 0 - 14 Å^-1^; k weight, 3; and Morlet function with κ=15, σ=1 was used as the mother wavelet to provide the overall distribution.

The different colors represent the height of the peak, which can not only distinguish the distance of the coordination atom (the bond length), but also distinguish the type of the coordination atom (the larger the atomic number, the farther to the right of the peak); The combination of Wavelet data and R-space fitting information can distinguish the coordination of samples well, and the Wavelet analysis can be more intuitive to see that the samples mainly contain Ni-O and Ni-M (M=Ni/Fe) coordination.


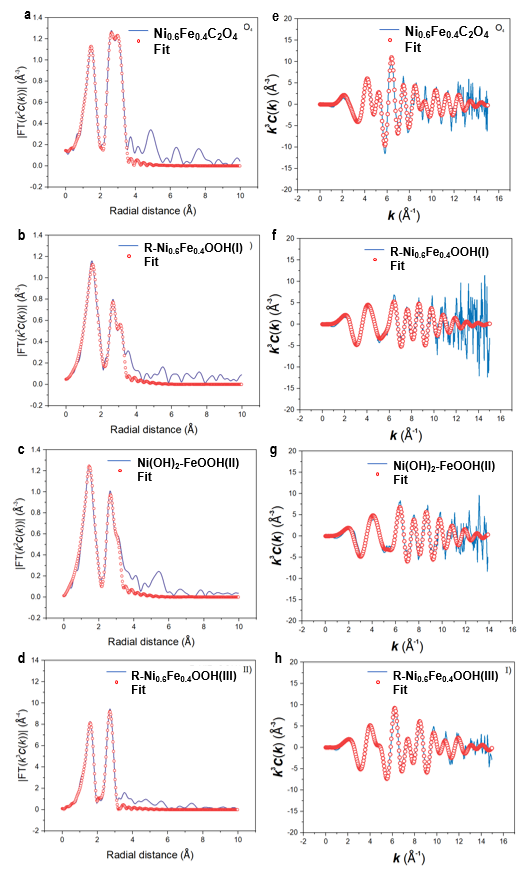


**Figure S16**. Fitting results of (a-h) Fe R/K-edge FT-EXAFS curves.

The combination of Wavelet data and R-space fitting information can distinguish the coordination of samples well, and the Wavelet analysis can be more intuitive to see that the samples mainly contain Fe-O and Fe-M (M=Ni/Fe) coordination.


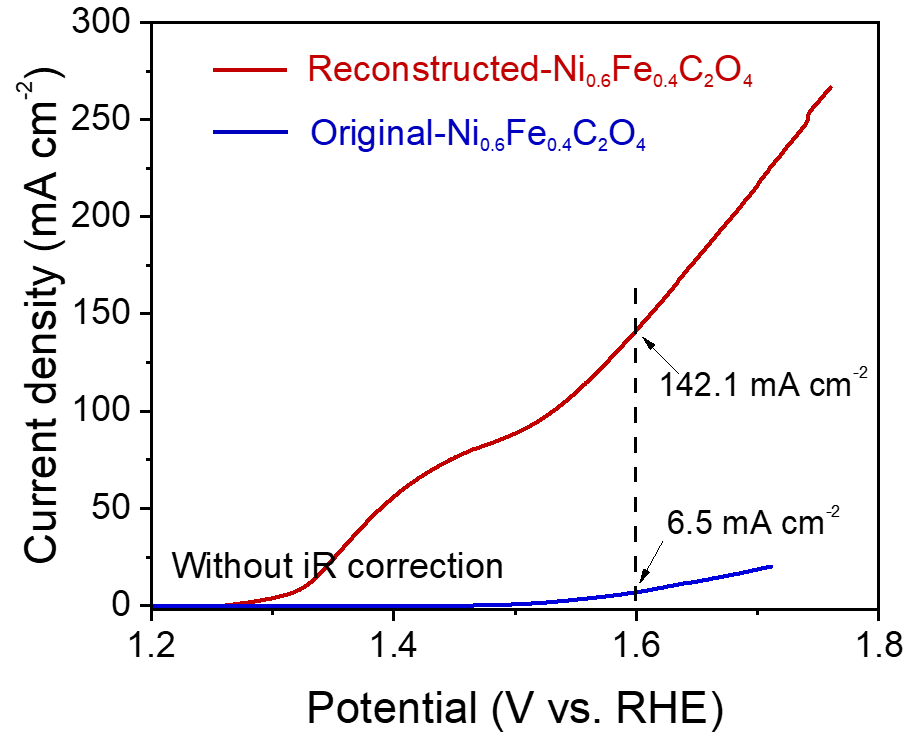


**Figure S17**. LSV (without iR correction) of original-Ni_0.6_Fe_0.4_C_2_O_4_ and reconstructed-Ni_0.6_Fe_0.4_C_2_O_4_ in 1M KOH

Considering the rapid reconstruction characteristics of Ni_0.6_Fe_0.4_C_2_O_4_ under oxidation potentials, we investigated the difference of OER activity between original-Ni_0.6_Fe_0.4_C_2_O_4_ and reconstructed-Ni_0.6_Fe_0.4_C_2_O_4_ under uncompensated conditions.


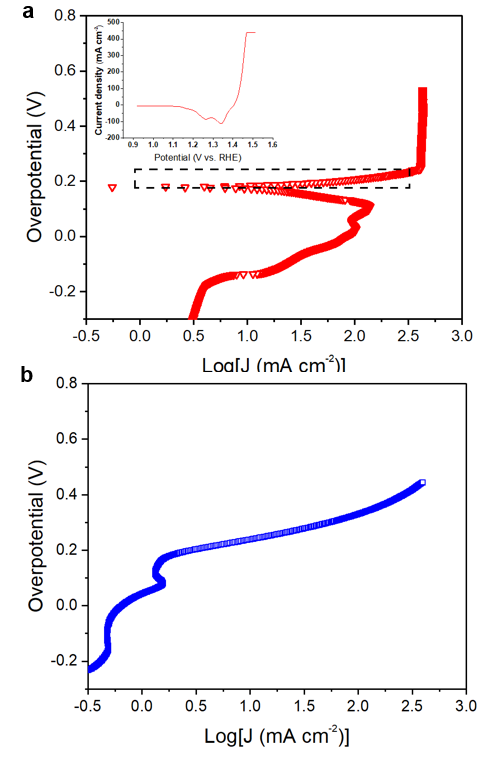


**Figure S18**. (a) Unfitted Tafel slope of R-Ni_0.6_Fe_0.4_OOH (the linear region of the OER is shown in the dashed box; illustration is the original OER-LSV of R-Ni_0.6_Fe_0.4_OOH and (b) RuO_2_.


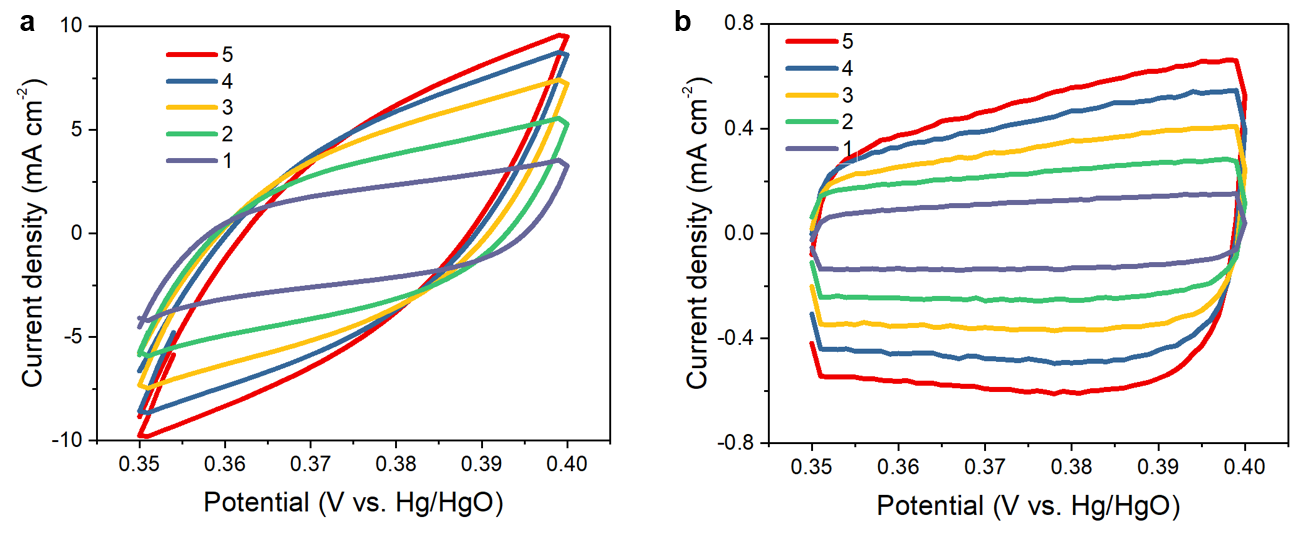


**Figure S19**. CV measurements at different scan rates. (a) R-Ni_0.6_Fe_0.4_OOH and (b) RuO_2_ in the potential range of 0.35–0.4 V vs. Hg/HgO at scan rates from 1 mV s^-1^ to 5 mV s^-1^.


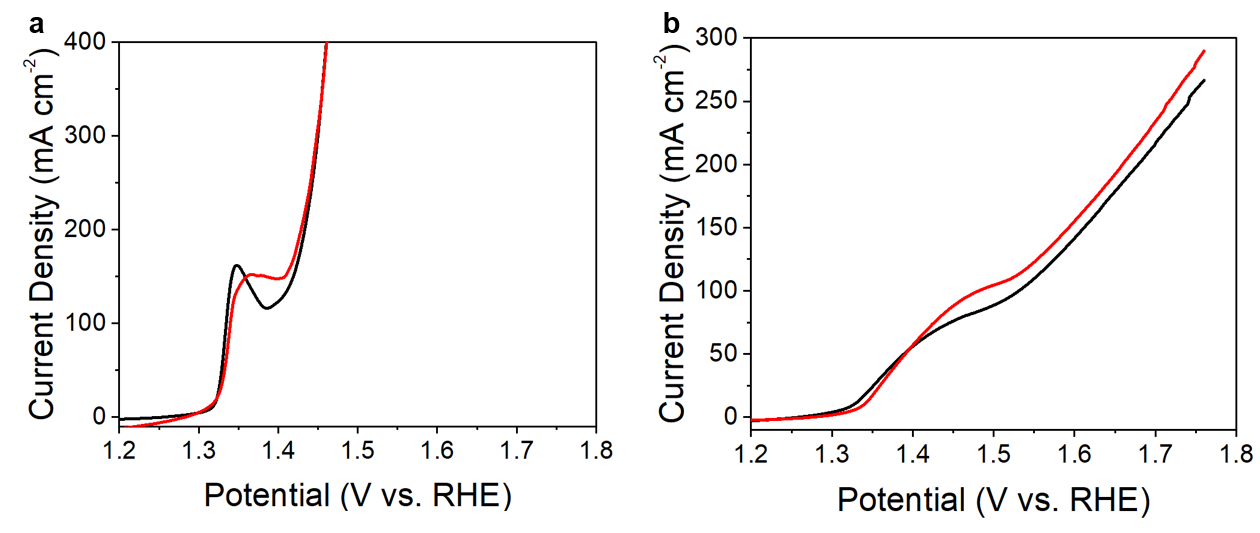


**Figure S20**. LSV plots. (a) with iR correction, (b) without iR correction (black curve is R-Ni_0.6_Fe_0.4_OOH, red curve is R-Ni_0.6_Fe_0.4_OOH after 250 h stability test at large current density).


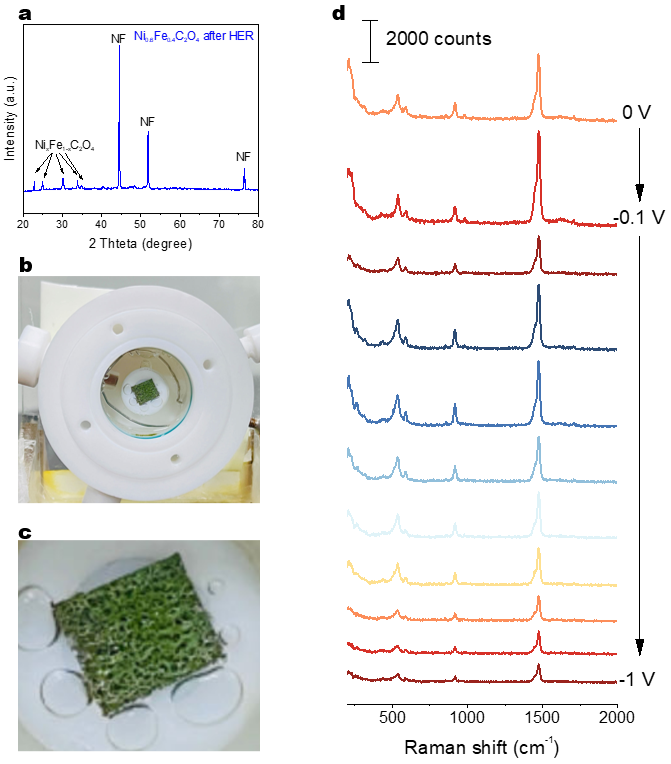


**Figure S21.** (a) XRD pattern of Ni_0.6_Fe_0.4_C_2_O_4_ after HER. (b-d) In situ Raman spectra and corresponding optical images collected on Ni_0.6_Fe_0.4_C_2_O_4_ electrocatalysts during HER.


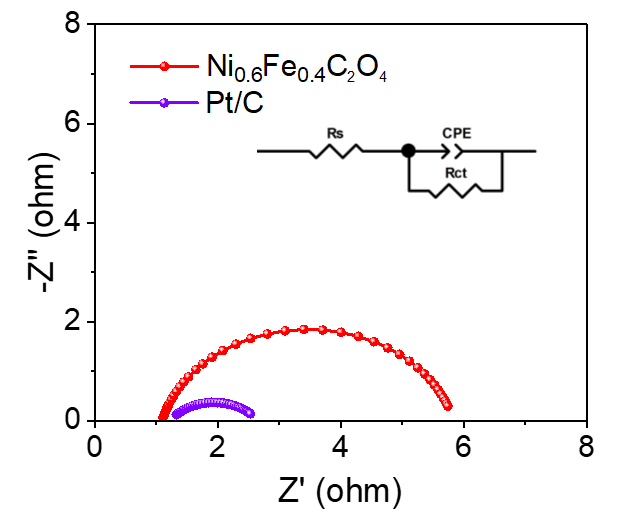


**Figure S22**. EIS plots of as-prepared Ni_0.6_Fe_0.4_C_2_O_4_ and Pt/C catalysts. Inset shows the equivalent circuit for EIS data fitting.


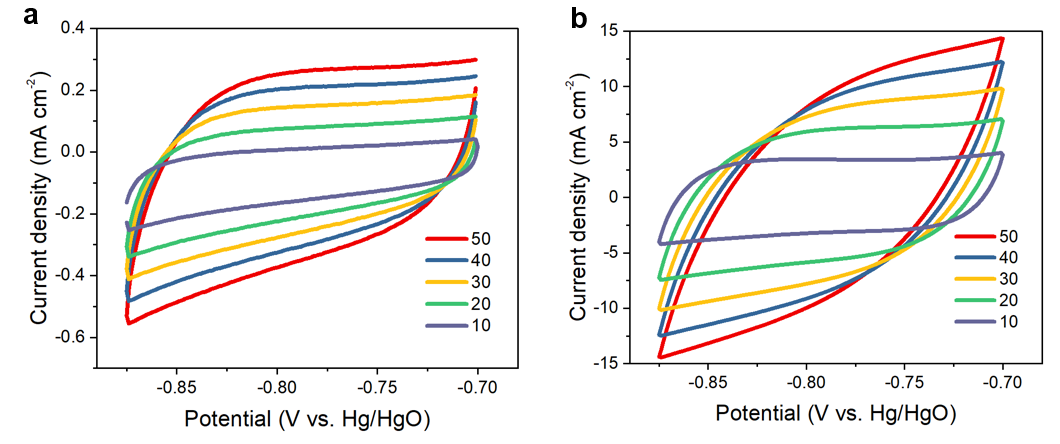


**Figure S23**. CV measurements at different scan rates. (a) Ni_0.6_Fe_0.4_C_2_O_4_ and (b) Pt/C in the potential range of –0.875 and –0.7 V vs. Hg/HgO at scan rates from 10 mV s^-1^ to 50 mV s^-1^.


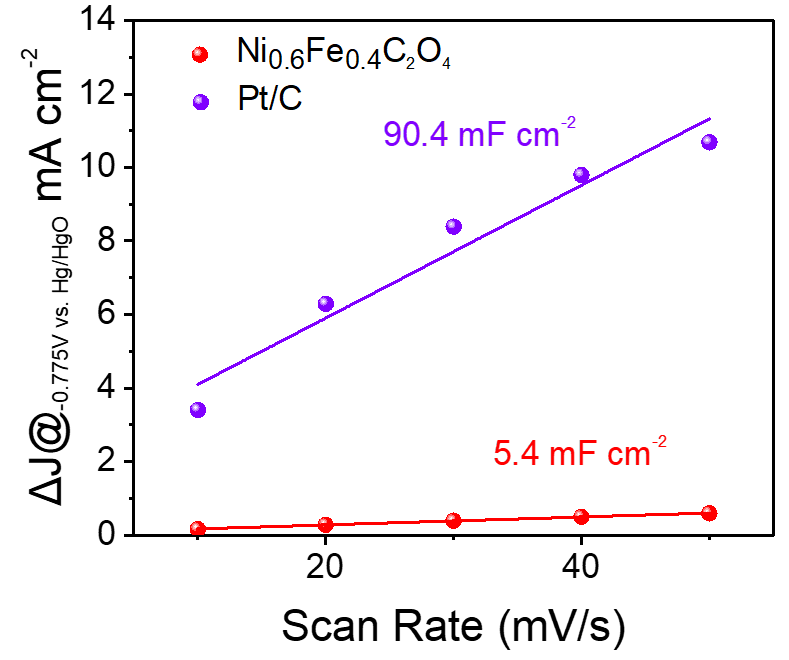


**Figure S24**. ECSA estimated by *C*_dl_ value.


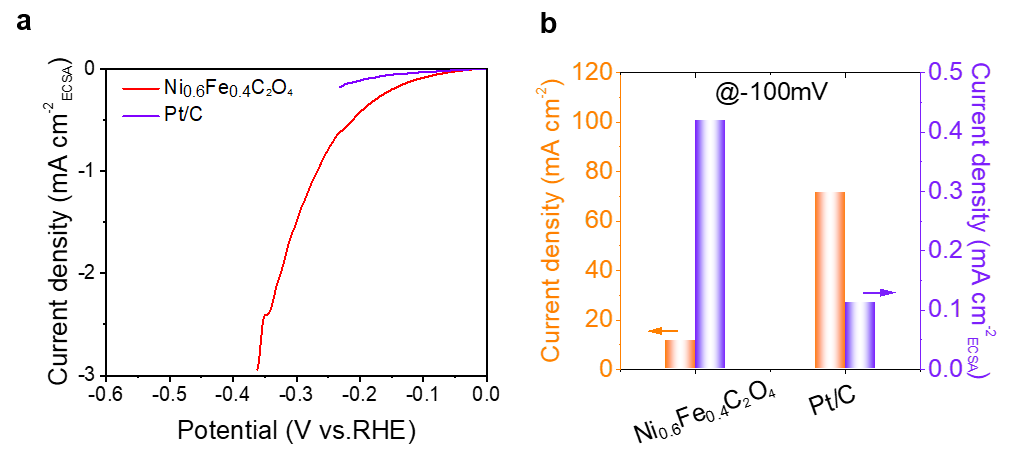


**Figure S25**. (a) ECSA normalized LSV curves of the Ni_0.6_Fe_0.4_C_2_O_4_ for HER in 1 M KOH. (b) HER geometrical activity and ECSA normalized activity of electrocatalysts at the potential of −100 mV.


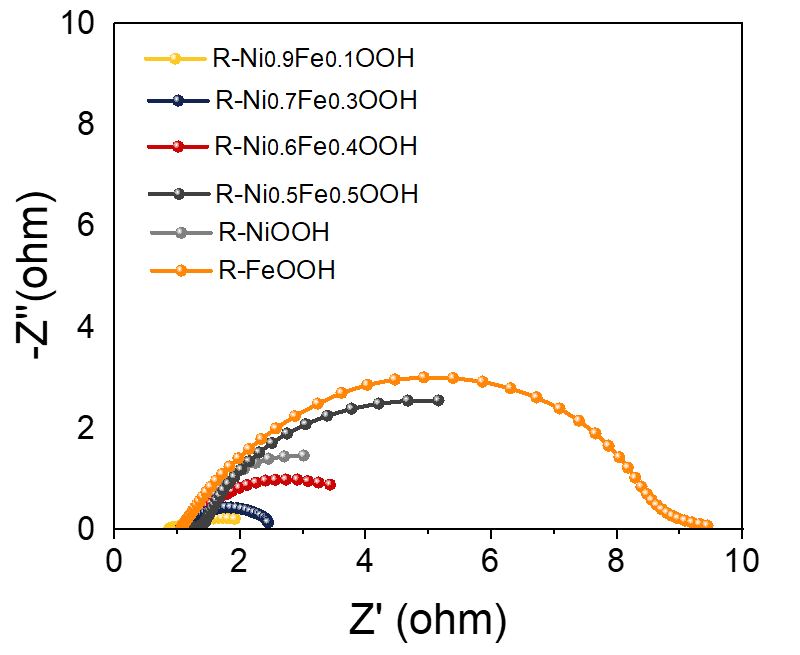


**Figure S26**. EIS plots of as-prepared R-Ni_x_Fe_1-x_OOH catalysts.

**Table S1.** EXAFS fitting parameters at the Ni K-edge for various samples.

| Sample | Path | CN | R（Å） | σ^2^（🞨10^-3^ Å^2^） | | E_0_（ev） | R-factor |
| --- | --- | --- | --- | --- | --- | --- | --- |
| Nifoil | Ni-M | 12 | 2.48±0.004 | 5.95±0.47 | -0.57±0.67 | | 0.0046 |
| Ni_0.6_Fe_0.4_C_2_O_4_ | Ni-O | 6.35±0.84 | 2.06±0.01 | 4.64±1.99 | 1.82 | | 0.019 |
|  | Ni-M | 7.33±0.47 | 2.97±0.01 | 1.54±2.32 |  | |  |
| R-Ni_0.6_Fe_0.4_OOH(Ⅰ) | Ni-O | 6.37±0.49 | 2.07±0.01 | 6.42±1.13 | 0.19 | | 0.004 |
|  | Ni-M | 7.24±1.07 | 3.13±0.01 | 7.29±1.22 |  | |  |
| Ni(OH)_2_-FeOOH(Ⅱ) | Ni-O | 7.04±0.74 | 2.06±0.01 | 8.71±1.65 | 1.01 | | 0.008 |
|  | Ni-M | 7.66±1.86 | 3.11±0.01 | 10.59±2.26 |  | |  |
| R-Ni_0.6_Fe_0.4_OOH(Ⅲ) | Ni-O | 6.63±0.52 | 2.04±0.01 | 9.12±1.28 | -1.33±0.85 | | 0.007 |
|  | Ni-M | 4.19±1.59 | 3.08±0.01 | 13.63±3.98 |  | |  |

*^a^CN*, coordination number; *^b^R*, distance between absorber and backscatter atoms; *^c^σ*^2^, Debye-Waller factor to account for both thermal and structural disorders; *^d^ΔE*_0_, inner potential correction; *R* factor indicates the goodness of the fit. S_0_^2^ was fixed to 0.82, according to the experimental EXAFS fit of Ni foil by fixing CN as the known crystallographic value. A reasonable range of EXAFS fitting parameters: 0.600 < *Ѕ*_0_^2^ < 1.000; *CN >* 0; *σ*^2^ > 0 Å^2^; |Δ*E*_0_| < 15 eV; *R* factor < 0.02.

**Table S2.** EXAFS fitting parameters at the Fe K-edge for various samples.

| Sample | Path | N | R（Å） | σ^2^（🞨10^-3^ Å^2^） | | E_0_（ev） | R-factor |
| --- | --- | --- | --- | --- | --- | --- | --- |
| Fefoil | Fe-Fe | 12 | 2.48±0.004 | 5.95±0.47 | -0.57±0.67 | | 0.0046 |
| Ni_0.6_Fe_0.4_C_2_O_4_ | Fe-O | 4.53±0.34 | 1.93±0.01 | 10.48±1.41 | 0.18 | | 0.002 |
|  | Fe-M | 4.15±1.42 | 2.94±0.01 | 10.03±2.60 |  |  |  |
| R-Ni_0.6_Fe_0.4_OOH(Ⅰ) | Fe-O | 5.65±0.22 | 2.01±0.02 | 1.52±3.33 |  | |  |
|  | Fe-M | 4.52±0.90 | 3.06±0.03 | 11.58±5.62 | -0.02 | | 0.011 |
| Ni(OH)_2_-FeOOH(Ⅱ) | Fe-O | 6.03±0.57 | 1.99±0.02 | 1.45±3.88 |  | |  |
|  | Fe-M | 5.11±0.45 | 3.05±0.03 | 12.29±6.11 | -3.25 | | 0.013 |
| R-Ni_0.6_Fe_0.4_OOH(Ⅲ) | Fe-O | 6.75±0.32 | 2.01±0.004 | 8.04±0.80 | 0.91 | | 0.002 |
|  | Fe-M | 7.35±0.59 | 3.08±0.004 | 8.54±0.80 |  | |  |

*^a^CN*, coordination number; *^b^R*, distance between absorber and backscatter atoms; *^c^σ*^2^, Debye-Waller factor to account for both thermal and structural disorders; *^d^ΔE*_0_, inner potential correction; *R* factor indicates the goodness of the fit. S_0_^2^ was fixed to 0.75, according to the experimental EXAFS fit of Fe foil by fixing CN as the known crystallographic value. A reasonable range of EXAFS fitting parameters: 0.600 < *Ѕ*_0_^2^ < 1.000; *CN >* 0; *σ*^2^ > 0 Å^2^; |Δ*E*_0_| < 15 eV; *R* factor < 0.02.

**References**

[1] B. Ravel, M. Newville, *J. Synchrotron Radiat.* **2005**, 12, 537.

[2] S. I. Zabinsky, J. J. Rehr, A. Ankudinov, R. C. Albers, M. J. Eller, *Physical Review B* **1995**, 52, 2995.

[3] H. Funke, A. C. Scheinost, M. Chukalina, *Physical Review B* **2005**, 71, 0.94110.

[4] X. Zhang, X. Zhang, H. Xu, Z. Wu, H. Wang, Y. Liang, *Adv. Funct. Mater.* **2017**, 27, 1606635.

[5] G. Kresse, J. Furthmüller, *Comput. Mater. Sci* **1996**, 6, 15.

[6] G. Kresse, D. Joubert, *Physical Review B* **1999**, 59, 1758.

[7] G. Kresse, J. Furthmüller, *Physical Review B* **1996**, 54, 11169.

[8] A. Peles, *J. Mater. Sci.* **2012**, 47, 7542.

[9] G. A. Sawatzky, J. W. Allen, *Phys. Rev. Lett.* **1984**, 53, 2339.
